# A product control framework for extracellular vesicle therapeutics: Quality attributes, stability, analytical procedures, and biodistribution

**DOI:** 10.1016/j.ijpx.2026.100650

**Published:** 2026-08-25

**Authors:** Hyunjun Moon, Suk Han Jung, Sungjoon Bae, Gyuri Bae, Areum Lee, Jin Hee Na, Jong-Ho Kim, Jeong Uk Choi, Sangmin Lee

**Affiliations:** a College of Pharmacy and Institute of Integrated Pharmaceutical Sciences, Kyung Hee University, Seoul 02447, Republic of Korea; b Department of Regulatory Science, Graduate School, Kyung Hee University, Seoul 02447, Republic of Korea; c Institute of Regulatory Innovation through Science (IRIS), Kyung Hee University, Seoul 02447, Republic of Korea; d Department of Fundamental Pharmaceutical Science, Graduate School, Kyung Hee University, Seoul 02447, Republic of Korea; e Department of Precision Medicine, Graduate School, Kyung Hee University, Seoul 02447, Republic of Korea

**Keywords:** Extracellular vesicles, Critical quality attributes, Potency, Stability, Analytical procedures, Biodistribution, Regulatory science

## Abstract

Extracellular vesicle (EV) therapeutics are being developed as native or minimally modified products and as engineered delivery systems, yet their composition and activity depend strongly on source cells, manufacturing, formulation, and storage. This review examines how established pharmaceutical and regulatory principles can be applied to EV product development in the United States, the European Union, Japan, and the Republic of Korea. We organize candidate quality attributes into four domains: identity, purity, potency, and safety-related quality attributes and controls. Criticality should be established through product-specific risk assessment rather than a fixed assay panel. We propose a three-axis framework for describing stability evidence by biological source, product state and storage, and downstream process sequence, supplemented by key study descriptors. Analytical procedures are evaluated according to the quality decisions they support in characterization, process control, release, stability, and comparability. For products with a complex or partly defined mechanism of action, we propose that a functional procedure anchor the potency strategy. For nonclinical biodistribution, we propose a modular framework from which blood or biofluid kinetics, longitudinal imaging, quantitative tissue analysis, and cellular or spatial localization can be selected. Interpretation depends on defining the measured entity and establishing the stability of product–label association. Although legal classifications differ across jurisdictions, the underlying scientific questions are similar. Near-term regulatory convergence should focus on common technical product descriptions, transparent CQA decisions, measurement comparability, and consistent reporting of stability and biodistribution studies. These frameworks support product-specific development rather than universal specifications or fixed acceptance criteria.

## Highlights

• Product definition precedes CQA and lifecycle control decisions.
• A four-domain framework supports product-specific CQA selection.
• A three-axis framework contextualizes EV stability evidence.
• Fit-for-purpose procedures are mapped to lifecycle quality decisions.
• Biodistribution interpretation begins with a defined measured entity.

## Graphical abstract

**Figure:**
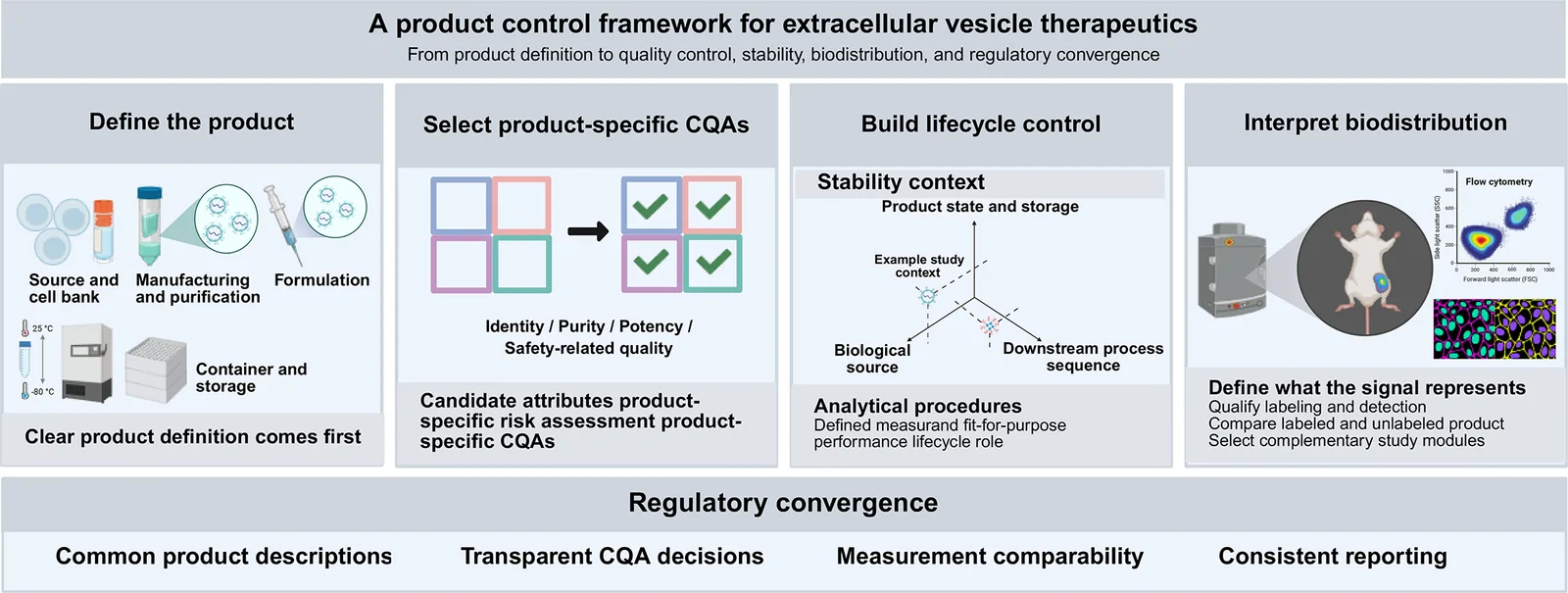

## 1 Introduction

Extracellular vesicles (EVs) are lipid bilayer-enclosed particles released by cells that transfer proteins, lipids, and nucleic acids (Welsh et al., 2024). Therapeutic development has followed two principal approaches. Native or minimally modified EV preparations are investigated for their intrinsic biological activity, whereas engineered EVs are designed to carry defined cargo or display functional surface molecules (Moon et al., 2025). Clinical interest has expanded rapidly, although most programs remain at an early stage. An analysis of ClinicalTrials.gov identified 355 studies of human-derived EVs initiated by December 31, 2025, including 148 interventional studies. Among these, 98 involved mesenchymal stromal cell-derived preparations, and most were early-phase investigations (Wei et al., 2026). Additionally, a systematic review reported encouraging early safety findings but substantial variation in manufacturing, study design, and safety reporting (Van Delen et al., 2024).

EV therapeutics do not constitute a single, well-defined pharmaceutical class. Each product comprises a heterogeneous population of cell-derived particles that may differ in size, membrane composition, surface proteins, and luminal cargo. These properties are affected by source cells and donors, cell-bank history, passage or population-doubling level, culture conditions, cell state, harvest methods, purification, engineering, formulation, and storage (Subcommittee on Therapeutic Products Based on Extracellular Vesicles (EVs) Including Exosomes, 2023; Takakura et al., 2024; Welsh et al., 2024). Particle concentration, mean size, and common EV-associated markers provide useful information, but they do not fully define product identity, purity, composition, or biological activity. Consequently, routine physical measurements may remain similar while composition or potency changes.

Therefore, manufacturing, analytical control, stability, and nonclinical evaluation should be considered as interconnected elements of development. Changes in oxygen tension, culture format, medium composition, passage level, or cell activation can alter EV composition and function without proportional changes in particle concentration or size distribution (Almeria et al., 2019; Kusuma et al., 2022; Tertel et al., 2023). Downstream processing can alter EV subpopulations and non-vesicular material. Formulation and container contact can further affect particle recovery, aggregation, membrane integrity, cargo retention, and potency during storage (Ahmadian et al., 2024; Gorgens et al., 2022; Trenkenschuh et al., 2022).

These dependencies create linked development decisions. The intended product population must be defined before unwanted subpopulations and process-related components can be classified as impurities. The potency strategy should be justified by the proposed mechanism of action and the available evidence linking the EV product as a whole to its intended biological activity, even when the contributions of individual components are not fully resolved. Each analytical procedure needs a defined role in characterization, in-process control, release, stability, or comparability. Shelf-life assignment requires evidence demonstrating that relevant physical, compositional, and functional attributes are maintained over time. Biodistribution interpretation requires a defined measured entity because an observed signal may represent product-associated material, released cargo, free label, or a label-derived material. Collectively, these decisions form a lifecycle control strategy.

MISEV2023 provides a widely used scientific framework for EV terminology, characterization, and reporting (Welsh et al., 2024). It recommends complementary evidence and cautions against assigning EV identity or function based on a single result. However, it was not developed as a regulatory specification and does not define release tests, acceptance criteria, stability protocols, comparability requirements, or approval pathways.

General pharmaceutical and biological product guidelines provide much of the regulatory basis for EV development. ICH Q6B describes identity, purity, potency, and biological product specifications (International Conference on Harmonisation of Technical Requirements for Registration of Pharmaceuticals for Human Use, 1999). ICH Q8(R2) and Q9(R1) support product-specific risk assessment and quality risk management (International Conference on Harmonisation of Technical Requirements for Registration of Pharmaceuticals for Human Use, 2009; International Council for Harmonisation of Technical Requirements for Pharmaceuticals for Human Use, 2023d). ICH Q5C and the draft consolidated Q1 guideline address stability (International Conference on Harmonisation of Technical Requirements for Registration of Pharmaceuticals for Human Use, 1995; International Council for Harmonisation of Technical Requirements for Pharmaceuticals for Human Use, 2025b). ICH Q14 and Q2(R2) address analytical procedure development and validation (International Council for Harmonisation of Technical Requirements for Pharmaceuticals for Human Use, 2023a, International Council for Harmonisation of Technical Requirements for Pharmaceuticals for Human Use, 2023e), while ICH Q5A(R2) and Q5E inform viral safety and manufacturing comparability (International Conference on Harmonisation of Technical Requirements for Registration of Pharmaceuticals for Human Use, 2004; International Council for Harmonisation of Technical Requirements for Pharmaceuticals for Human Use, 2023b). ICH S12 can inform nonclinical biodistribution study design, although its formal scope is gene therapy products (International Council for Harmonisation of Technical Requirements for Pharmaceuticals for Human Use, 2023c). The principal challenge lies in applying these principles to a heterogeneous particle product whose composition and function depend on source, process, formulation, and product design (Lee et al., 2026).

Regulatory classification also differs among regions. The United States applies existing drug and biological product frameworks without dedicated EV development guidance. In the European Union, advanced therapy medicinal product status is determined by the final product. Japan has issued an EV-specific scientific report without creating a separate statutory category. The Republic of Korea regulates EV therapy products as biological products and has issued dedicated nonbinding guidance (Committee for Advanced Therapies, 2026; European Commission, 2009; European Medicines Agency, 2018, European Medicines Agency, 2025, European Medicines Agency, 2026; European Parliament and Council of the European Union, 2007; National Institute of Food and Drug Safety Evaluation, 2024; Subcommittee on Therapeutic Products Based on Extracellular Vesicles (EVs) Including Exosomes, 2023; U.S. Food and Drug Administration, 2019, U.S. Food and Drug Administration, 2023). These differences affect legal pathways and regulatory interactions, but the main scientific questions remain product definition, manufacturing consistency, potency, stability, safety, and biodistribution.

Recent publications have reviewed multinational regulation, clinical development, product quality, therapeutic applications, storage, and preservation (Ahmadian et al., 2024; Chaudhari et al., 2026; Li et al., 2025; Van Delen et al., 2024; Verma and Arora, 2025; Wei et al., 2026; Xu et al., 2025). The PMDA Science Board report and related article provide detailed quality and safety considerations (Subcommittee on Therapeutic Products Based on Extracellular Vesicles (EVs) Including Exosomes, 2023; Takakura et al., 2024). However, a remaining need is a product-level framework that connects critical quality attribute (CQA) decisions with stability, analytical procedure selection, comparability, and biodistribution interpretation.

This review addresses that need from a pharmaceutical regulatory science perspective. We organize candidate quality attributes into identity, purity, potency, and safety-related quality. We then describe the context required to interpret stability evidence, assign analytical procedures according to their role in the control strategy, and propose a modular approach to nonclinical biodistribution. These frameworks support product-specific decisions rather than universal specifications, fixed assay panels, or common acceptance criteria. This review also identified areas where scientific expectations could converge despite differences in legal classifications.

### 1.1 Scope and review approach

This targeted narrative review focused on native and engineered EV preparations intended for therapeutic administration. It includes products in which the EV population, associated cargo, engineered surface component, or a combination of these features contributes to the intended activity. Mammalian cell-derived products receive primary attention because they dominate current clinical development and are the principal focus of available regulatory documents (National Institute of Food and Drug Safety Evaluation, 2024; Subcommittee on Therapeutic Products Based on Extracellular Vesicles (EVs) Including Exosomes, 2023; Takakura et al., 2024; Van Delen et al., 2024; Wei et al., 2026).

Plant-derived, milk-derived, and bacterial vesicles are discussed only when they illustrate source-specific quality or safety issues. Diagnostic and biomarker applications, unpurified conditioned media, cell secretomes, cell lysates, synthetic EV mimetics, and cosmetic products were outside the main scope, except where they help clarify a regulatory boundary or analytical limitation.

Public regulatory documents and peer-reviewed literature available through June 2026 were reviewed. Regulatory sources included documents from ICH, FDA, EMA, PMDA, MFDS, and the European Union. Scientific publications were identified through targeted PubMed searches and reference-list screening focused on EV manufacturing, product quality, potency, stability, analytical procedures, biodistribution, and regulatory development. Primary regulatory documents and original experimental studies were prioritized, whereas reviews were used to identify broader evidence patterns and additional studies. No meta-analysis was performed.

The review did not capture confidential scientific advice, unpublished sponsor-agency interactions, or internal review practices. A targeted search may also have missed relevant publications. Most clinical programs were at an early stage, and product-specific acceptance criteria were rarely public. Therefore the proposed frameworks organize current evidence and will require further evaluation across products, laboratories, and development stages. They should not be interpreted as regulatory requirements.

**Fig. 1:**
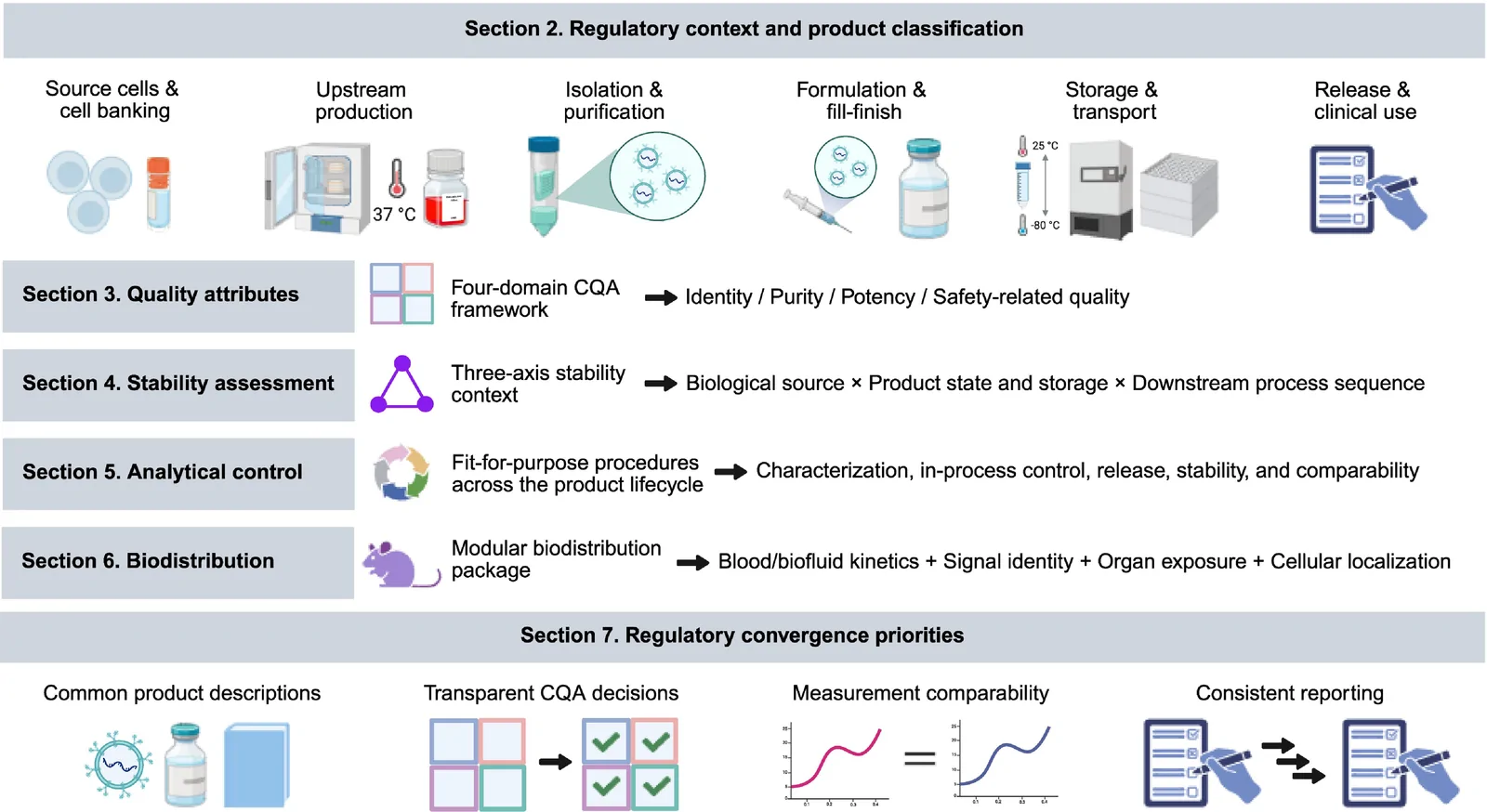
Organization of the review across the extracellular vesicle therapeutic product lifecycle. The upper row shows development from source-cell and cell-bank selection to product release and clinical use. 2, 3, 4, 5, 6, 7 address regulatory classification, quality attributes, stability, analytical control, biodistribution, and regulatory convergence. The four lower boxes summarize the main frameworks proposed in this review. The diagram is conceptual and does not prescribe a universal manufacturing sequence, assay panel, or development pathway.

The review follows the EV product lifecycle. Section 2 covers regulatory classification. 3, 4, 5, 6 address quality attributes, stability, analytical control, and biodistribution. Section 7 identifies priorities for regulatory convergence. Fig. 1 summarizes these relationships.

## 2 Regulatory landscape and product classification of EV therapeutics

EV therapeutic products do not fall within a single regulatory category across jurisdictions. Classification depends on the final product, including the source and modification of the producer cells, the nature and function of introduced cargo, and the proposed mechanism of action. Consequently, products based on similar EV platforms may follow different development pathways.

**Fig. 2:**
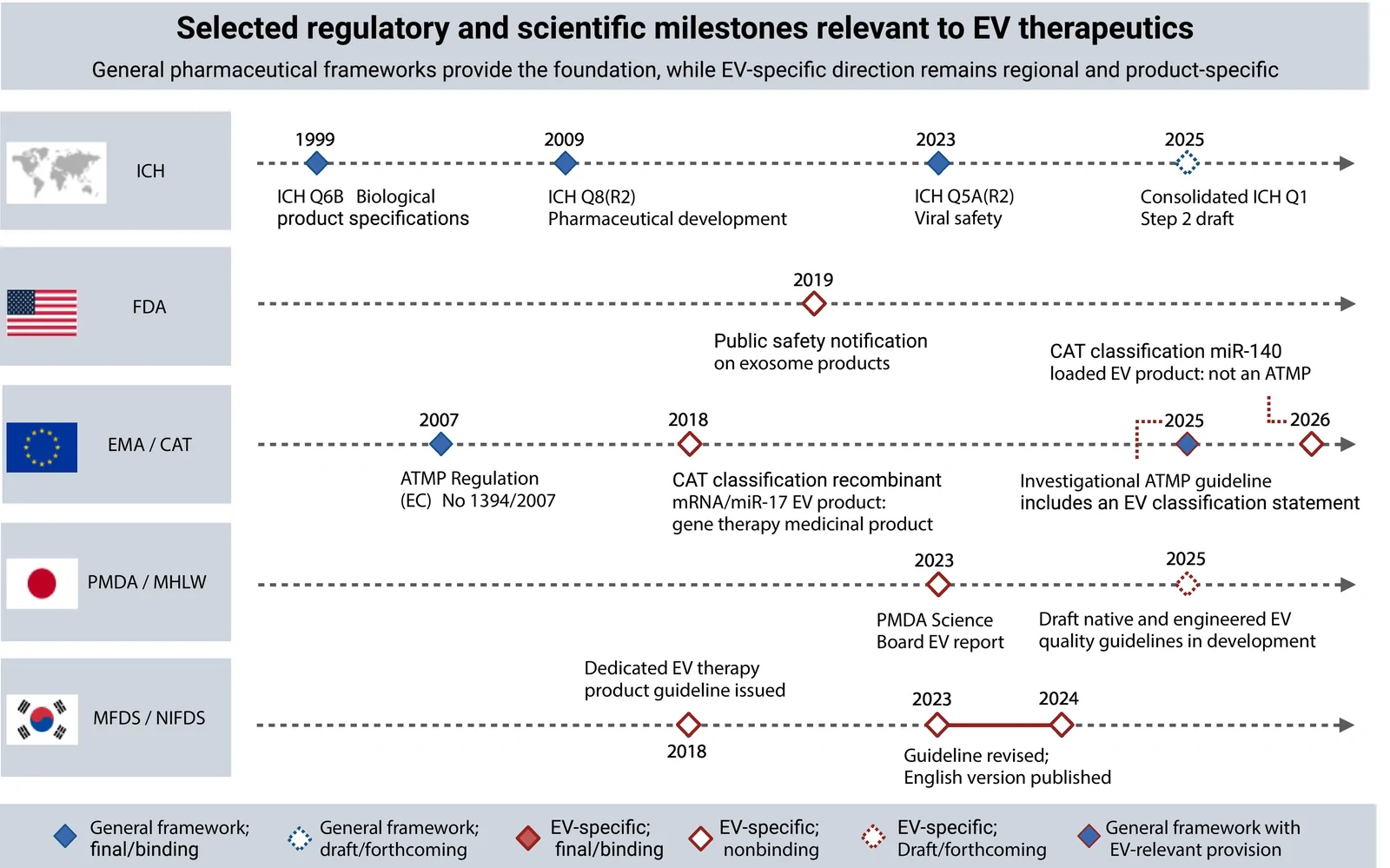
Selected regulatory and scientific milestones relevant to extracellular vesicle therapeutics. The timeline summarizes foundational ICH guidelines and EV-related actions in the United States, European Union, Japan, and Republic of Korea. Marker color indicates scope: blue denotes general pharmaceutical frameworks, and red denotes EV-specific milestones. Marker style indicates document status: solid, open, and dashed markers denote final or binding, nonbinding, and draft or forthcoming documents, respectively. A separate blue marker identifies a general framework that contains an EV-relevant provision (the 2025 EMA investigational ATMP guideline). Status is current through June 2026. (For interpretation of the references to color in this figure legend, the reader is referred to the web version of this article.)

Across the four jurisdictions, the underlying scientific questions are broadly similar, but the legal classification and EV-specific guidance differ. The United States applies existing drug and biological product requirements. The European Union determines advanced therapy medicinal product (ATMP) status based on the final product. Japan has issued an EV-specific report and is preparing EV-specific quality guidelines. The Republic of Korea regulates EV therapy products as biological products under dedicated nonbinding guidance. Fig. 2 summarizes the selected regulatory and scientific milestones.

### 2.1 Regulatory treatment by jurisdiction

Legal classification determines the development pathway and applicable guidance, but does not define the analytical package required for an individual product. Table 1 compares the four jurisdictions and their practical implications.

**Table 1:** Regulatory treatment of extracellular vesicle therapeutic products in the United States, European Union, Japan, and the Republic of Korea.

Authority and region	Principal regulatory basis	Current classification position	EV-specific regulatory material	Practical implications for development
FDA (CBER), United States	Federal Food, Drug, and Cosmetic Act, Public Health Service Act, 21 CFR Part 312, and applicable guidance for biological products	Therapeutic exosome products are regulated as drugs and biological products. Clinical investigation requires an effective IND, and marketing requires an approved BLA.	No dedicated guidance for EV therapeutic development. Public materials mainly address safety concerns and enforcement involving unapproved exosome products.	Existing guidance should be selected based on the final product, including its source, engineering, cargo, and mechanism of action. Early FDA interaction is important.
EMA and CAT, European Union	Directive 2001/83/EC, Regulation (EC) No 1394/2007, and applicable guidance for biological medicinal products and ATMPs	Product-specific. ATMPs are biological medicinal products; the two categories are not mutually exclusive. EVs and cellular fragments of human-cell origin do not themselves meet the current ATMP definition. An EV-based final product may nevertheless qualify as a gene therapy medicinal product if the statutory criteria are met. Non-ATMP EV medicinal products remain under the general EU medicinal-product framework.	No dedicated EV quality guideline. The 2025 EMA investigational ATMP guideline includes an EV classification statement. The optional CAT classification procedure provides a nonbinding scientific recommendation.	Classification depends on the statutory characteristics of the final product, not the term ‘EV’ itself. Borderline cases should be clarified early; outcomes should not be extrapolated across products.
PMDA and MHLW, Japan	The Pharmaceuticals and Medical Devices Act and existing guidance were selected according to the defined product and its risks.	No EV-specific statutory category has been established. Applicable requirements are selected case by case according to product composition, producer cells, engineering, and mechanism of action.	The PMDA Science Board report was issued in 2023. In 2025, PMDA reported the preparation of separate draft quality guidelines for native and engineered EV products, with MHLW publication planned for FY2026 after public consultation.	The report provides detailed EV-specific scientific considerations. The legal pathway, specifications, and acceptance criteria remain product-specific. Draft documents should not be cited as final guidance.
MFDS and NIFDS, Republic of Korea	Pharmaceutical Affairs Act and regulations for biological products. Additional provisions may apply to genetically modified products.	EV therapy products are not cell therapy products because they do not contain living cells. They are regulated as biological products. Gene therapy product criteria may apply when genetic modification is used.	A dedicated nonbinding guideline was issued in 2018, revised in 2023, and published in English in 2024.	The guideline provides an EV-specific development structure. Analytical methods, acceptance criteria, and study designs still require product-specific justification.

**Table notes** 1. Classification describes the applicable legal or regulatory pathway. It does not define the analytical specification for an individual EV product. 2. Products with different source cells, cargos, engineering methods, or mechanisms of action may receive different classifications within the same jurisdiction. 3. Guidance documents, scientific reports, and classification recommendations do not have the same legal status as statutes, regulations, or approval decisions. 4. Regulatory status is current through June 2026. 5. IND, Investigational New Drug application; BLA, Biologics License Application; ATMP, advanced therapy medicinal product; CAT, Committee for Advanced Therapies; CBER, Center for Biologics Evaluation and Research; MFDS, Ministry of Food and Drug Safety; NIFDS, National Institute of Food and Drug Safety Evaluation; PMDA, Pharmaceuticals and Medical Devices Agency; MHLW, Ministry of Health, Labour and Welfare. 6. Key sources are FDA (U.S. Food and Drug Administration, 2019, U.S. Food and Drug Administration, 2023), EMA and European Union sources (Committee for Advanced Therapies, 2026, European Commission, 2009, European Parliament and Council of the European Union, 2007, European Medicines Agency, 2018, European Medicines Agency, 2025, European Medicines Agency, 2026), PMDA and MHLW sources (Kuribayashi, 2025; Subcommittee on Therapeutic Products Based on Extracellular Vesicles (EVs) Including Exosomes, 2023; Takakura et al., 2024), and MFDS and NIFDS sources (National Institute of Food and Drug Safety Evaluation, 2024).

#### 2.1.1 United States

In the United States, exosome products intended to treat diseases are regulated as drugs under the Federal Food, Drug, and Cosmetic Act and as biological products under the Public Health Service Act (U.S. Food and Drug Administration, 2019, U.S. Food and Drug Administration, 2023). Clinical administration requires an effective Investigational New Drug application, and marketing requires an approved Biologics License Application (U.S. Food and Drug Administration, 2023).

The FDA has issued no dedicated EV development guidance. Its EV-specific public materials primarily address unapproved products and do not provide a development-stage CMC framework (U.S. Food and Drug Administration, 2019, U.S. Food and Drug Administration, 2023).

Therefore, sponsors must apply existing biological product guidance according to the characteristics of the final product, including considerations related to cell substrates, viral safety, genetic modification, potency, and specifications. Early interaction with the FDA is particularly important when the active component, dose unit, potency strategy, comparability plan, or biodistribution method are not well established. The absence of EV-specific guidance does not reduce the evidence expected for an IND or BLA.

#### 2.1.2 European Union

The European Union regulates ATMPs under Regulation (EC) No 1394/2007 and Directive 2001/83/EC (European Commission, 2009; European Parliament and Council of the European Union, 2007). Under EU legislation, ATMPs are biological medicinal products; therefore, “biological medicinal product” and “ATMP” are not mutually exclusive categories. ATMP status is determined by whether the final product meets the statutory definition of a gene therapy medicinal product, somatic cell therapy medicinal product, tissue-engineered product, or combined ATMP. The 2025 EMA guideline states that extracellular vesicles and cellular fragments originating from human cells do not themselves fulfill the current ATMP definition (European Medicines Agency, 2025). Because EV preparations are acellular, they do not meet the cell- or tissue-based definitions of somatic cell therapy medicinal products or tissue-engineered products. Nevertheless, an EV-based final product may qualify as a gene therapy medicinal product when it contains a recombinant nucleic acid active substance and its therapeutic effect is directly related to that sequence or its expression product. EV medicinal products outside ATMP scope remain subject to the general EU medicinal-product framework. Classification therefore depends on the statutory characteristics of the final product rather than on the term “EV” itself.

Developers may request an optional, nonbinding ATMP classification recommendation from the Committee for Advanced Therapies (European Medicines Agency, 2026). This process can clarify borderline cases but does not replace national decisions or define the complete quality and nonclinical package.

Published outcomes show the product-specific approach. In 2018, exosomes carrying recombinant mRNA encoding the cystic fibrosis transmembrane conductance regulator protein and microRNA-17 were classified as gene therapy medicinal products (European Medicines Agency, 2018). In March 2026, the CAT concluded that EVs derived from hTERT-expressing Wharton jelly mesenchymal stromal cells and loaded with microRNA-140 did not meet the ATMP definition (Committee for Advanced Therapies, 2026). Therefore classification cannot be inferred from the term EV, the presence of nucleic acid cargo, or the use of a genetically modified producer cell alone. A non-ATMP conclusion does not place a product outside medicinal product regulation, and classification outcomes should not be extrapolated to products with different sources, cargos, manufacturing processes, or mechanisms of action.

#### 2.1.3 Japan

Japan has not created a separate statutory category for EV therapeutics. In January 2023, the PMDA Science Board issued an EV-specific report, followed by a related peer-reviewed article in 2024 (Subcommittee on Therapeutic Products Based on Extracellular Vesicles (EVs) Including Exosomes, 2023; Takakura et al., 2024). The nonbinding report addresses source-cell control, manufacturing, characterization, purity, potency, viral safety, stability, pharmacology, biodistribution, toxicity, and first-in-human development. Native and engineered products are considered separately where their risks differ.

In December 2025, PMDA reported that separate draft quality guidelines for native and engineered EV products were being prepared, with MHLW publication planned for FY2026 following public consultation (Kuribayashi, 2025). Until publication, these documents should be described as drafts or forthcoming.

The Science Board report does not assign EV products as a class to regenerative medicine or gene therapy pathways. Applicable requirements must be selected case by case according to product composition, producer cells, introduced components, mechanism of action, and safety risks. The report provides detailed EV-specific scientific directions but does not prescribe universal assays or numerical acceptance criteria. Therefore, product-specific consultation remains necessary.

#### 2.1.4 Republic of Korea

The MFDS guideline defines an EV therapy product as a pharmaceutical product manufactured by isolating and purifying EVs produced by living cells. It excludes unpurified cell suspensions, cell lysates, and vesicle mimetics produced *via* physical disruption (National Institute of Food and Drug Safety Evaluation, 2024). Given that EV therapy products do not contain living cells, they are not classified as cell therapy products. They are regulated as biological products, although gene therapy product requirements may also apply when genetic modification is involved (National Institute of Food and Drug Safety Evaluation, 2024).

The nonbinding guideline was issued in 2018, revised in 2023, and published in English in 2024. It covers starting materials, cell banks, manufacturing, characterization, stability, nonclinical studies, and clinical development, and links potency testing to the proposed mechanism of action (National Institute of Food and Drug Safety Evaluation, 2024).

The guideline provides an EV-specific development framework but does not establish universal specifications or numerical limits. Analytical procedures, acceptance criteria, dose units, potency assays, stability panels, and nonclinical study designs still require product-specific justification.

### 2.2 Areas of regulatory convergence and remaining gaps

Despite differences in legal classifications, all four jurisdictions address similar scientific issues related to source control, manufacturing, identity, purity, potency, safety, stability, and nonclinical distribution. The primary differences lie in how these expectations enter formal development pathways.

These approaches should not be ranked by maturity. Dedicated guidance can improve predictability but may remain broad when late-stage experience is limited. Case-by-case review provides flexibility but can create uncertainty. A classification procedure can clarify the legal pathway without resolving detailed quality and manufacturing questions.

International harmonisation remains limited. The ICH has not issued an EV-specific guideline. The consolidated ICH Q1 guideline reached Step 2 in April 2025 and remained under revision through June 2026, with Step 4 adoption planned for November 2026 (ICH Q1 Expert Working Group, 2026; International Council for Harmonisation of Technical Requirements for Pharmaceuticals for Human Use, 2025b). Although the draft covers biological products and ATMPs, it does not specifically address EV therapeutics. MISEV2023 provides scientific guidance on terminology and characterization but does not define release specifications or approval requirements (Welsh et al., 2024). Additionally, the 2025 ICH Cell and Gene Therapy Discussion Group report noted that legal classification and nomenclature may remain region-specific even when scientific expectations can be aligned (ICH Cell and Gene Therapy Discussion Group, 2025).

The major gaps are technical rather than purely legal. They include defining the active product, selecting CQAs and dose units, developing a relevant potency strategy, establishing comparability after manufacturing changes, identifying stability-indicating attributes, qualifying biodistribution methods, and developing reference systems that support measurement comparability. Addressing these challenges will require shared technical language and transparent product-specific justification, rather than a common legal category or assay panel.

## 3 Quality attributes and CQA selection for extracellular vesicle therapeutics

### 3.1 Rationale for product-specific CQA selection

ICH Q8(R2) defines a CQA as a physical, chemical, biological, or microbiological property that should remain within an appropriate limit, range, or distribution to ensure product quality (International Conference on Harmonisation of Technical Requirements for Registration of Pharmaceuticals for Human Use, 2009). Not every measurable attribute is critical. Criticality should be established for the defined product by linking attribute variability to the quality target product profile and potential effects on safety or efficacy. ICH Q6B provides a useful starting point by organizing biological product specifications around identity, purity, potency, quantity, and general tests (International Conference on Harmonisation of Technical Requirements for Registration of Pharmaceuticals for Human Use, 1999), although its examples focus primarily on well-defined proteins.

Applying these principles to EV therapeutics requires additional interpretation because the active material consists of a heterogeneous particle population. Particles within the same batch may differ in size, membrane composition, surface proteins, and luminal cargo. These properties depend on the source cell, donor, culture conditions, cell state, purification, formulation, and storage (Subcommittee on Therapeutic Products Based on Extracellular Vesicles (EVs) Including Exosomes, 2023; Takakura et al., 2024; Welsh et al., 2024). Consequently, similar particle counts and size distributions may mask changes in composition or activity, whereas losses in recoverable particles may not produce proportional reductions in potency.

Product identity and dose assignment are also less straightforward. No marker is present on all EV populations, and commonly measured tetraspanins do not establish source identity or therapeutic function (Subcommittee on Therapeutic Products Based on Extracellular Vesicles (EVs) Including Exosomes, 2023; Takakura et al., 2024; Welsh et al., 2024). Particle number, total protein, active cargo, and biological activity may each provide dose or normalization information, but none is universally suitable. Therefore, the dose unit should be justified based on the manufacturing process, product composition, proposed mechanism of action, and analytical capabilities (Subcommittee on Therapeutic Products Based on Extracellular Vesicles (EVs) Including Exosomes, 2023; Takakura et al., 2024).

A candidate quality attribute is considered during development before criticality has been established. ICH Q8(R2) and Q11 use potential CQAs to support risk assessment and process development (International Conference on Harmonisation of Technical Requirements for Registration of Pharmaceuticals for Human Use, 2009, International Conference on Harmonisation of Technical Requirements for Registration of Pharmaceuticals for Human Use, 2012). We organize candidate attributes into identity, purity, potency, and safety-related quality. This author-developed structure is informed by ICH Q6B, ICH Q5E, MISEV2023, and current PMDA and MFDS materials (International Conference on Harmonisation of Technical Requirements for Registration of Pharmaceuticals for Human Use, 1999, International Conference on Harmonisation of Technical Requirements for Registration of Pharmaceuticals for Human Use, 2004; National Institute of Food and Drug Safety Evaluation, 2024; Subcommittee on Therapeutic Products Based on Extracellular Vesicles (EVs) Including Exosomes, 2023; Takakura et al., 2024; Welsh et al., 2024). It is not an official ICH classification.

Quantity is treated as a cross-cutting variable because particle number, protein mass, cargo amount, and biological activity can describe dose or normalize another result. Safety-related quality also distinguishes measurable product attributes from lifecycle controls. Some risks can be monitored in the final product, whereas others are more effectively managed through donor and cell-bank qualification, raw-material control, process validation, or nonclinical testing.

Each candidate attribute should undergo product-specific risk assessment before being designated as a CQA. The control strategy should define whether an attribute is used for characterization, in-process control, release, stability, or comparability. Stability is not treated as a fifth domain; rather, it reflects time-dependent change across all four domains. A single attribute may support multiple decisions. For example, particle size distribution contributes to physical characterization and may also detect aggregation during stability testing.

**Fig. 3:**
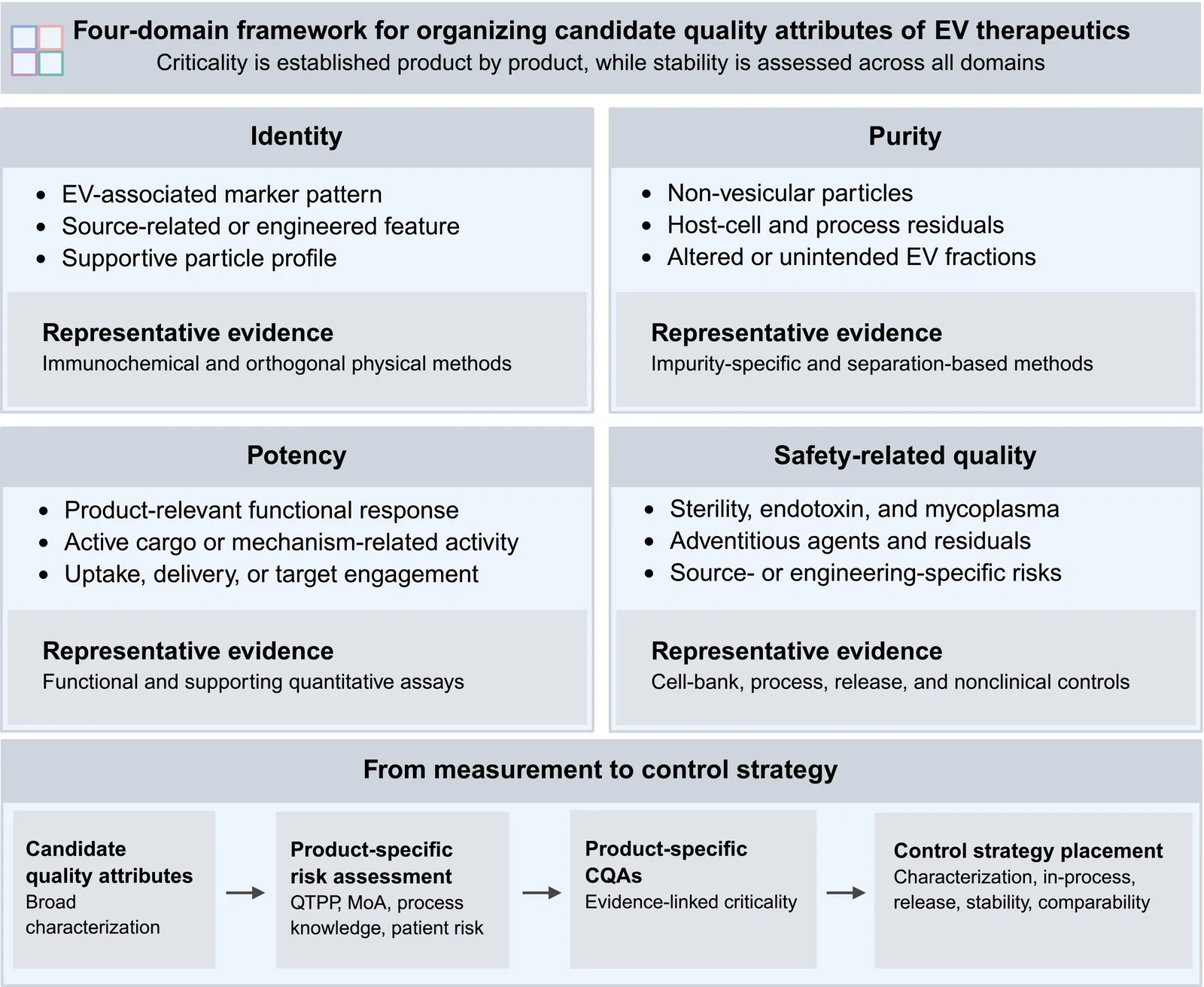
Four-domain framework for organizing candidate quality attributes of extracellular vesicle therapeutic products. The framework groups candidate attributes into identity, purity, potency, and safety-related quality. Criticality is established for each product through risk assessment and evidence linking attribute variability to safety or efficacy. The domains are not mutually exclusive, and one attribute may support more than one quality decision. Quantity is treated as a cross-cutting dose or normalization variable, while stability is assessed as time-dependent change across all four domains. Safety-related quality links measurable product attributes with controls applied to source materials, manufacturing, release, and nonclinical development. The framework is author-proposed and does not define a universal assay panel or release specification.

Candidate attributes should not be confused with routine release tests. ICH Q6B distinguishes comprehensive product characterization from the smaller set of tests included in a specification (International Conference on Harmonisation of Technical Requirements for Registration of Pharmaceuticals for Human Use, 1999). A release panel should confirm routine product quality rather than reproduce the full characterization package. Fig. 3 illustrates how candidate attributes are assessed and placed within a product-specific control strategy.

### 3.2 Four-domain framework for organizing candidate quality attributes

The four domains organize related but different quality questions. Identity asks whether the material is the intended EV product. Purity asks which unintended product- or process-related components are present. Potency addresses biological activity relevant to the proposed mechanism of action. Safety-related quality addresses the product attributes and lifecycle controls required to manage microbial, viral, chemical, and source-related risks. Although conceptually distinct, the domains are not mutually exclusive. Representative candidate attributes and analytical approaches are summarized in Table 2.

**Table 2:** Four-domain framework for organizing candidate quality attributes of extracellular vesicle therapeutic products.

Domain	Core question	Representative candidate attributes	Representative analytical approaches	Key interpretation
**Identity**	Is the material the intended EV product?	EV-associated membrane and luminal proteins, source-related markers, defining engineered features, association of cargo with the EV fraction, morphology, and size profile	Capillary immunoassay, ELISA, high-sensitivity flow cytometry, single-particle imaging, NTA, TRPS, and electron microscopy	No universal EV marker exists. Marker results should be interpreted with complementary physical evidence. Particle counting and sizing do not establish identity alone(Subcommittee on Therapeutic Products Based on Extracellular Vesicles (EVs) Including Exosomes, 2023; Takakura et al., 2024; Welsh et al., 2024).
**Purity**	Which unintended product- or process-related components are present?	Non-vesicular particles, soluble proteins, lipoproteins, altered or unintended EV fractions, host-cell proteins and DNA, medium components, process residuals, and free cargo	Impurity-specific immunoassays, qPCR or digital PCR, chromatography, asymmetric flow field-flow fractionation, targeted proteomics, and particle-to-protein ratio as a comparative process metric	Purity depends on the intended product definition. An EV subpopulation is not an impurity solely because it differs in size or marker profile. The particle-to-protein ratio is not a universal release criterion (International Conference on Harmonisation of Technical Requirements for Registration of Pharmaceuticals for Human Use, 1999; Subcommittee on Therapeutic Products Based on Extracellular Vesicles (EVs) Including Exosomes, 2023; Takakura et al., 2024; Webber and Clayton, 2013; Welsh et al., 2024).
**Potency**	Does the product show the intended biological activity?	Product-relevant functional response, active cargo or ligand, enzyme or receptor activity, intracellular delivery, target engagement, and downstream signaling	Cell-based bioassays, reporter assays, biochemical activity assays, immunoassays, nucleic acid assays, flow cytometry, and imaging-based procedures.	For products with a complex or partly defined mechanism of action, at least one functional assay should anchor the potency strategy. A surrogate procedure requires a demonstrated relationship with biological activity (Gimona et al., 2021; International Conference on Harmonisation of Technical Requirements for Registration of Pharmaceuticals for Human Use, 1999; Subcommittee on Therapeutic Products Based on Extracellular Vesicles (EVs) Including Exosomes, 2023; Takakura et al., 2024; U.S. Food and Drug Administration, 2011).
**Safety-related quality**	Which product attributes and lifecycle controls are needed to manage safety risks?	Microbial contamination, bacterial endotoxin, mycoplasma, adventitious agents, residual DNA or vector, process residuals, procoagulant activity, and immunogenic or tumor-associated material.	Compendial microbiological procedures, recombinant Factor C (rFc) or LAL assay, PCR, sequencing, *in vitro* virus assays, coagulation assays, immunoassays, and targeted molecular assays.	Safety control extends across source qualification, cell banks, raw materials, manufacturing, release, and nonclinical testing. Not every safety-related attribute is a final-product CQA or routine release test (International Council for Harmonisation of Technical Requirements for Pharmaceuticals for Human Use, 2023b; National Institute of Food and Drug Safety Evaluation, 2024; Subcommittee on Therapeutic Products Based on Extracellular Vesicles (EVs) Including Exosomes, 2023; Takakura et al., 2024).

**Table notes** 1. A candidate quality attribute is an attribute considered during development before its criticality has been established. Designation as a CQA and placement within the control strategy require product-specific justification. 2. The four domains are organizational and are not mutually exclusive. A single attribute may support multiple quality decisions. 3. Particle number, protein mass, active cargo content, and biological activity are cross-cutting dose or normalization variables rather than a separate quality domain. 4. Stability is assessed by monitoring time-dependent changes in relevant attributes across all four domains. 5. Safety-related quality includes both measurable product attributes and controls applied to source materials, cell banks, manufacturing, release, and nonclinical development. 6. MISEV2023 provides a scientific framework for EV characterization but does not establish regulatory release specifications. 7. The framework is an author-developed adaptation informed by ICH Q8(R2), Q11, Q6B, Q5E, MISEV2023, and current PMDA and MFDS materials (International Conference on Harmonisation of Technical Requirements for Registration of Pharmaceuticals for Human Use, 1999, International Conference on Harmonisation of Technical Requirements for Registration of Pharmaceuticals for Human Use, 2004, International Conference on Harmonisation of Technical Requirements for Registration of Pharmaceuticals for Human Use, 2009, International Conference on Harmonisation of Technical Requirements for Registration of Pharmaceuticals for Human Use, 2012; National Institute of Food and Drug Safety Evaluation, 2024; Subcommittee on Therapeutic Products Based on Extracellular Vesicles (EVs) Including Exosomes, 2023; Takakura et al., 2024; Welsh et al., 2024). 8. AF4, asymmetrical flow field-flow fractionation; LAL, limulus amebocyte lysate; NTA, nanoparticle tracking analysis; TRPS, tunable resistive pulse sensing.

#### 3.2.1 Identity

Identity testing should establish vesicular nature, intended biological source, and any defining engineered feature. MISEV2023 recommends complementary evidence because no single marker or method is sufficient (Welsh et al., 2024). EV-associated proteins including CD9, CD63, CD81, ALIX, TSG101, and syntenin can support identity, but their abundance varies by source and EV subtype. Accordingly, the same panel should not be applied universally. Markers of intracellular compartments and likely co-isolated materials should also reflect the source and manufacturing process.

Source identity should be supported by one or more product-relevant markers or compositional features that distinguish the intended population from unrelated material. For example, dendritic cell-derived EVs may require assessment of antigen-presentation molecules. Products generated from genetically modified cells may require the measurement of an introduced surface protein or cargo together with evidence that the engineered feature is associated with the EV fraction. Detection of cargo in the bulk formulation does not establish an EV association.

Particle concentration, size distribution, morphology, and surface charge provide supportive physical characterization. Particle concentration may contribute to dose assignment, whereas changes in size distribution may indicate aggregation or other product alterations. However, these measurements do not establish identity on their own because NTA, TRPS, and DLS can also detect non-vesicular particles within their respective measurement ranges (Subcommittee on Therapeutic Products Based on Extracellular Vesicles (EVs) Including Exosomes, 2023; Takakura et al., 2024; Welsh et al., 2024).

During product definition, the identity package should combine product-specific biochemical or immunochemical evidence with physical characterization. Single-particle methods may be useful for describing marker-positive subpopulations and marker co-detection patterns. Not every method is appropriate for routine release testing. Western blotting, for example, may remain useful for extended characterization, whereas routine identity procedures should provide adequate specificity, reproducibility, and transferability. Whether qualitative or quantitative, the selected test should distinguish the intended product from relevant alternatives (International Conference on Harmonisation of Technical Requirements for Registration of Pharmaceuticals for Human Use, 1999).

#### 3.2.2 Purity

Purity is meaningful only after the intended product population has been defined. An EV preparation may contain intended EVs, acceptable product-related variants, altered or degraded EVs, unintended EV subpopulations, non-vesicular particles, and process-related impurities. These categories should be defined before analytical limits are established. An EV subpopulation should not be classified as an impurity solely because it differs in size or marker profile. Its classification should reflect its effect on product activity and safety (International Conference on Harmonisation of Technical Requirements for Registration of Pharmaceuticals for Human Use, 1999; Subcommittee on Therapeutic Products Based on Extracellular Vesicles (EVs) Including Exosomes, 2023; Takakura et al., 2024).

Non-vesicular materials may include soluble protein complexes, lipoproteins, protein aggregates, nucleic acid complexes, and particles introduced through raw materials or consumables. Process-related impurities may include residual host-cell proteins and DNA, culture-medium components, serum-derived particles, antibiotics, nucleases, affinity ligands, chromatography residuals, filtration-related material, and purification or formulation reagents. The relevant profile depends on both the upstream culture system and downstream process.

Microbial and viral contaminants may share physical properties with EVs and can be difficult to remove once contamination occurs (International Council for Harmonisation of Technical Requirements for Pharmaceuticals for Human Use, 2023b; Subcommittee on Therapeutic Products Based on Extracellular Vesicles (EVs) Including Exosomes, 2023; Takakura et al., 2024). These risks should be managed through source, process, and product controls within the safety strategy described in Section 3.2.4. Purity assessment should focus on the intended particle population and defined product-related and process-related impurities.

The particle-to-protein ratio can serve as an in-process indicator or comparative metric within a defined manufacturing platform, but it is not a universal purity specification. The result depends on the source material, particle-counting method, protein assay, formulation, and abundance of EV-associated proteins. Thresholds proposed in early studies were derived from specific samples and isolation procedures and were not established as regulatory acceptance criteria (Webber and Clayton, 2013). Therefore, purity assessment requires impurity-specific and separation-based evidence.

#### 3.2.3 Potency

ICH Q6B defines potency as a quantitative measure of biological activity and generally expects a relevant assay within the biological product specification (International Conference on Harmonisation of Technical Requirements for Registration of Pharmaceuticals for Human Use, 1999). The appropriate procedure depends on the active components and the extent to which the mechanism of action is understood. For EV products with a complex or partly defined mechanism, we propose that at least one functional assay should anchor the potency strategy. The procedure should provide a quantitative result over a defined range, demonstrate dose-response behavior where relevant, distinguish acceptable product from materially altered material, and exhibit precision appropriate to its intended use (Gimona et al., 2021; Takakura et al., 2024; U.S. Food and Drug Administration, 2011).

Native EV products may act through multiple components and pathways; therefore, a single assay may not capture all relevant activities. A potency matrix can combine a primary functional assay with supporting measurements of cargo, surface ligands, enzyme activity, receptor interaction, cellular association, or downstream signaling. Each procedure should have a defined role. Multiple weakly related assays do not compensate for a poor relationship between selected readouts and product function.

A functional assay should measure a response relevant to the proposed mechanism of action or intended indication. Depending on the product, this may involve inhibition of inflammatory signaling, modulation of immune-cell activity, restoration of barrier function, promotion of tissue repair, target-cell killing, or delivery of a functional nucleic acid. Appropriate controls should distinguish EV-mediated activity from effects attributable to soluble proteins, residual medium components, or other non-vesicular materials.

For engineered EVs carrying a defined cargo, cargo amount and integrity provide important potency-related information but may be insufficient when activity also depends on EV association, membrane integrity, cell binding, uptake, intracellular release, or target engagement. A surrogate procedure should replace functional assessment only after a reliable relationship with biological activity has been demonstrated across representative and altered lots.

Potency assay development should begin early and progress from exploratory mechanism-related procedures to qualified and, where appropriate, validated lot-release methods. Stability-indicating capability should also be assessed. A potency method that cannot distinguish intact products from materials altered during storage has limited value for shelf-life assignment.

#### 3.2.4 Safety-related product attributes and lifecycle controls

Safety control begins with the source cells and manufacturing process and cannot rely solely on final-vial testing. Routine final-product controls may include sterility, bacterial endotoxins, mycoplasma, residual host-cell DNA and protein, and relevant process residuals. Adventitious agent control often spans donor or source qualification, cell-bank testing, raw-material control, manufacturing, and product testing. Genetically modified cells require additional control of the expression construct, vector-related sequences, and unintended products associated with the modification (International Council for Harmonisation of Technical Requirements for Pharmaceuticals for Human Use, 2023b; Subcommittee on Therapeutic Products Based on Extracellular Vesicles (EVs) Including Exosomes, 2023; Takakura et al., 2024).

Viral safety requires particular attention because EVs and many viruses overlap in size and may behave similarly during filtration and chromatography. Conventional viral inactivation or clearance steps may damage the EV product. ICH Q5A(R2) was not written specifically for all EV therapeutics, but its principles can inform cell-substrate qualification, raw-material control, virus testing, and evaluation of viral clearance where feasible (ICH Cell and Gene Therapy Discussion Group, 2025; International Council for Harmonisation of Technical Requirements for Pharmaceuticals for Human Use, 2023b). The overall strategy should reflect the source cell, manufacturing process, and the extent to which clearance or inactivation measures can be introduced without unacceptable effects on product quality.

Additional safety-related attributes should reflect the source, route, dose, and product design. Examples include procoagulant activity in tissue factor-positive preparations, immunogenic surface antigens, residual vector or transgene products, unintended bioactive cargo, and tumor-associated material from immortalized or tumorigenic source cells. Products derived from induced pluripotent stem cells may also require the assessment of residual pluripotency-associated or tumorigenicity-related materials.

Not every safety risk should become a final-product CQA or routine release test. A measurable attribute may be designated as a CQA when its variability is linked to patient risk and can be controlled through a suitable procedure. Other risks may be managed more effectively through cell-bank qualification, raw-material specifications, process validation, extended characterization, or nonclinical testing. Therefore, the safety-related quality domain links safety-relevant product attributes with lifecycle controls rather than defining a fixed test panel. Across all four domains, the task is to select a justified set of attributes and controls for manufacturing, release, stability, and comparability.

### 3.3 Adapting the framework to source and product design

The framework should be adapted to a defined product rather than applied as a fixed assay panel. Source cells are important determinants of EV composition and activity, but donor characteristics, tissue origin, cell-bank history, passage or population-doubling level, culture medium, oxygen tension, cell state, purification, formulation, and engineering also affect the final preparation (Subcommittee on Therapeutic Products Based on Extracellular Vesicles (EVs) Including Exosomes, 2023; Takakura et al., 2024; Welsh et al., 2024). Therefore, candidate attributes should be selected with reference to source, process history, and product design.

MSC-derived EVs demonstrate why a source-cell label alone is insufficient. Hypoxic culture increased angiogenic activity without major changes in particle concentration, size, or most surface markers (Almeria et al., 2019). Similarly, two-dimensional and three-dimensional culture systems produced preparations with similar particle counts and tetraspanin profiles but different proteomic and immunomodulatory properties (Kusuma et al., 2022). Physical measurements and common markers therefore cannot establish consistency alone. The control strategy should address donor and tissue origin, cell-bank characteristics, passage, senescence, medium, oxygen tension, preconditioning, and culture format. A product-relevant potency assay should assess upstream changes. Tissue factor activity, immunogenic components, and residual culture-supplement material may also require control (Subcommittee on Therapeutic Products Based on Extracellular Vesicles (EVs) Including Exosomes, 2023; Takakura et al., 2024).

Immortalized cells and induced pluripotent stem cell-derived MSCs can provide greater expansion capacity and a more continuous cell-bank supply but do not ensure functional homogeneity. EV preparations from different batches have differed in their ability to modulate allogeneic immune responses *in vitro* (Tertel et al., 2023). Therefore, an expandable source does not remove the need for lot-specific functional assessment. Additional controls may include genetic stability, the reprogramming or immortalization system, residual vector-related sequences, and comparability following cell-bank expansion or replacement (Subcommittee on Therapeutic Products Based on Extracellular Vesicles (EVs) Including Exosomes, 2023; Takakura et al., 2024; Tertel et al., 2023).

Immune cell-derived EVs require greater emphasis on source-cell activation and immune function. For dendritic cell-derived products, maturation state, antigen loading, major histocompatibility complex molecules, costimulatory proteins, and active ligands may contribute to identity or potency. In a phase II study of interferon-γ-matured dendritic cell-derived exosomes in non-small cell lung cancer, major histocompatibility complex class II molecules and tetraspanins were used as release parameters, but the primary clinical endpoint was not met (Besse et al., 2016). Increased NKp30-dependent natural killer cell activity in some patients further supports linking phenotypic markers with a functional immune assay. For T cell-derived or natural killer cell-derived EVs, relevant attributes may include cytotoxic cargo, target-cell recognition, killing activity, residual activation cytokines, and unintended immune stimulation (Subcommittee on Therapeutic Products Based on Extracellular Vesicles (EVs) Including Exosomes, 2023; Takakura et al., 2024).

Tumor cell-derived EVs require particular attention to safety. They may contain intended tumor antigens along with oncogenic nucleic acids, immunosuppressive molecules, or other materials that could support tumor growth or immune escape. When developed as vaccines, the product definition should distinguish components supporting the intended response from those that may contribute to unwanted activity. Broader molecular characterization may be needed than for products from non-tumorigenic cells. Source-cell carryover, tumor-associated cargo, residual nucleic acids, and unintended immunomodulatory activity should be addressed within the purity and safety strategy (Subcommittee on Therapeutic Products Based on Extracellular Vesicles (EVs) Including Exosomes, 2023; Takakura et al., 2024).

Engineering is a product-design feature, not a separate biological source. It may be introduced through producer-cell modification or through cargo loading and surface modification after isolation (Asadujjaman et al., 2025). Candidate attributes include the identity and amount of the introduced feature, loading efficiency, unassociated cargo, EV association, cargo integrity, ligand density or accessibility, and lot-to-lot consistency. For nucleic acid-loaded products, sequence identity, structural integrity, protection from degradation, intracellular delivery, and target engagement may contribute to potency (Park et al., 2025). EVs carrying KRAS^G12D-targeting RNA illustrate that activity depends on both cargo and carrier properties (Kamerkar et al., 2017). Cargo amount should not replace a functional potency assay unless its relationship with activity has been demonstrated across representative lots.

Furthermore, non-mammalian vesicle products require source-specific adaptation. Plant- and milk-derived vesicles may require controls for source authentication, microbial burden, allergenic components, and non-vesicular materials derived from the source matrix. Bacterial outer membrane vesicles possess distinct structural and immunological properties and should not be evaluated using an unmodified mammalian EV panel. Lipopolysaccharide may contribute to intended immunostimulatory activity while also limiting tolerability. In one study, its removal increased the maximum tolerated dose of engineered outer membrane vesicles by more than 25-fold (Chen et al., 2025). Therefore, lipopolysaccharide amount and activity should be evaluated as product-specific attributes rather than solely as generic contaminant measurements.

Source- and design-specific attributes do not constitute a fifth domain. Instead, they should be assigned to identity, purity, potency, or safety-related quality according to their role in the defined product. The same source cell can generate different products when culture, purification, formulation, or engineering are altered. Comparability assessments following donor, cell-bank, process, or engineering changes should therefore evaluate product-relevant function alongside physical and compositional attributes. These same considerations should guide the selection of the stability-indicating attributes discussed in Section 4.

## 4 Stability assessment of extracellular vesicle therapeutics

### 4.1 Regulatory basis and product-specific stability considerations

Stability assessment of EV therapeutics should be grounded in the current framework for biological products. ICH Q5C remains the principal final guideline for biological product stability, while ICH Q1A(R2) provides relevant general principles (International Conference on Harmonisation of Technical Requirements for Registration of Pharmaceuticals for Human Use, 1995, International Conference on Harmonisation of Technical Requirements for Registration of Pharmaceuticals for Human Use, 2003). The consolidated ICH Q1 guideline reached Step 2 in April 2025 and remained a draft as of June 2026 (ICH Q1 Expert Working Group, 2026; International Council for Harmonisation of Technical Requirements for Pharmaceuticals for Human Use, 2025b). The current work plan targets Step 3 sign-off and Step 4 adoption in November 2026. If adopted, the consolidated guideline is intended to replace the existing Q1 series and ICH Q5C. Although it covers biological products, frozen products, and advanced therapy medicinal products, it does not specifically address EV therapeutics.

Long-term studies under the proposed storage condition remain the primary basis for shelf-life assignment. Accelerated and stress studies can support formulation and analytical procedure development, comparability, and investigation of degradation pathways, but they should not replace long-term data for biological products. A formal shelf-life program should use representative batches in the proposed container-closure system and evaluate a product-specific set of stability-indicating attributes (International Conference on Harmonisation of Technical Requirements for Registration of Pharmaceuticals for Human Use, 1995, International Conference on Harmonisation of Technical Requirements for Registration of Pharmaceuticals for Human Use, 1999; International Council for Harmonisation of Technical Requirements for Pharmaceuticals for Human Use, 2025b). The number and scale of batches should reflect the development stage and intended regulatory use. For marketing applications, current ICH Q5C expectations include stability information from at least three representative final-container batches (International Conference on Harmonisation of Technical Requirements for Registration of Pharmaceuticals for Human Use, 1995).

EV products present several potential failure modes. Storage may result in adsorption to contact surfaces, aggregation, particle fusion, membrane disruption, alteration of surface proteins, degradation or release of luminal cargo, and loss of biological activity (Bosch et al., 2016; Gelibter et al., 2022; Gorgens et al., 2022; Levy et al., 2023; Lyu et al., 2022). These changes may occur independently and at different rates. Particle concentration and size distribution may remain unchanged while cargo integrity or potency declines. Conversely, reduced particle recovery may reflect container adsorption or sample handling rather than product degradation. No single measurement is therefore sufficient to establish stability.

A stability protocol should define the product presentation, formulation, concentration, fill volume, container closure system, storage and shipping conditions, freeze-thaw history, thawing procedure, post-thaw hold time, and administration-related handling. The analytical panel should be designed to monitor relevant physical, compositional, and functional changes. Separate accelerated, stress, shipping, freeze-thaw, and in-use studies should be used to identify failure modes and support formulation, packaging, and handling instructions.

### 4.2 Storage temperature and product state

Storage at −80 °C is common for frozen liquid EV preparations, but it is neither a universal standard nor a complete stability strategy. Reported outcomes vary according to source, purification, concentration, formulation, container, storage duration, and analytical procedure (Ahmadian et al., 2024; Gelibter et al., 2022; Gorgens et al., 2022; Levy et al., 2023; Lyu et al., 2022). Therefore, storage conditions should be justified using the defined product in its intended presentation.

Görgens et al. compared EV preparations from several cell sources under different buffer and temperature conditions (Gorgens et al., 2022). Highly purified EVs stored in phosphate-buffered saline (PBS) revealed significant losses in recoverable particles, including during storage at −80 °C. A formulation containing PBS, HEPES, human serum albumin, and trehalose improved recovery during short- and long-term storage. Dilution in PBS also reduced recovery within minutes. Therefore, storage temperature cannot be evaluated independently of formulation, concentration, and sample handling.

Other studies have reached different conclusions under different conditions. Gelibter et al. observed time-dependent changes in particle concentration, size distribution, purity, and zeta potential (Gelibter et al., 2022). Levy et al. found that native and cargo-loaded MSC-derived EVs differed in the stability of functional endpoints (Levy et al., 2023). Lyu et al. reported the preservation of selected physical and functional attributes for four weeks under optimized frozen or lyophilized conditions (Lyu et al., 2022). Collectively, these findings show that stability cannot be inferred from a single physical attribute or extrapolated across products without careful consideration of study context.

Refrigerated storage may support short-term handling or post-thaw use when supported by product-specific data. Storage at −20 °C should also be evaluated experimentally because published studies have reported retained activity and losses in particles or increases in aggregation (Gelibter et al., 2022; Levy et al., 2023). Claims for room-temperature storage of liquid products require evidence obtained using the final formulation and container, including functional and physical endpoints. Formal stability studies of frozen liquid products should use the intended formulation, concentration, fill volume, primary container, and closure. Supporting studies should evaluate temperature excursions, transport, post-thaw storage, and the maximum interval between thawing and administration. Storage statements should specify defined temperature ranges rather than terms such as room temperature (International Council for Harmonisation of Technical Requirements for Pharmaceuticals for Human Use, 2025b).

### 4.3 Freeze-thaw, shipping, and in-use stability

Freeze-thaw exposure should be minimized through product and supply-chain design. Single-use clinical vials and separate aliquots for release, stability, and retention testing can reduce unnecessary refreezing. The permitted number of freeze-thaw cycles should be established experimentally. Study protocol should define freezing rate, storage duration before thawing, thawing temperature and time, mixing, post-thaw hold time, concentration, fill volume, and container type. Testing should include particle recovery, size distribution and aggregation, product-specific identity or composition markers, cargo integrity where relevant, and potency.

The effects of freeze-thaw cycling depend on both formulation and product characteristics. Bosch et al. found that 25 mM trehalose reduced aggregation and preserved selected biological activity of pancreatic beta-cell-derived EV-like particles (Bosch et al., 2016). Görgens et al. reported improved recovery across repeated freeze-thaw cycles in PBS-HAT compared with PBS (Gorgens et al., 2022). Gelibter et al. observed changes in concentration, size distribution, and membrane-related properties after freeze-thaw exposure (Gelibter et al., 2022). These results support product-specific cycle limits rather than applying a uniform limit across EV products.

Shipping studies should reproduce the intended distribution configuration and account for temperature excursions, vibration, agitation, container orientation, exposure to dry ice or refrigerants where relevant, and the maximum expected transport duration with an appropriate safety margin. Samples should be evaluated after the complete simulation and, where relevant, after subsequent storage or thawing.

In-use studies should address all manipulations occurring after removal from the labeled storage condition, including thawing, dilution, transfer to syringes or infusion bags, passage through tubing or filters, light exposure, and holding before administration. Compatibility with diluents and administration materials should be evaluated because adsorption, aggregation, or loss of delivered dose can occur after the product leaves the primary container. The proposed in-use period should be supported by a justified subset of stability-indicating procedures, including potency when meaningful changes may occur.

### 4.4 Formulation, stabilizing excipients, and container closure

Formulation is a major determinant of EV stability. It can affect membrane hydration, electrostatic interactions, aggregation, surface adsorption, cargo retention, and biological activity. Relevant variables include buffer composition, pH, ionic strength, osmolality, EV concentration, stabilizing excipients, surfactants, protein excipients, antioxidants, and other formulation components.

PBS should not be regarded as a default pharmaceutical formulation simply because it is widely used in laboratory studies. Görgens et al. reported improved particle recovery after adding HEPES, human serum albumin, and trehalose (Gorgens et al., 2022), and Bosch et al. reported a protective effect of trehalose during freeze-thaw exposure (Bosch et al., 2016). Collectively, these studies support systematic formulation screening but do not establish a universal excipient combination.

Each excipient should have a clearly defined function and a justified concentration. Safety, route compatibility, analytical interference, and effects on product quality should also be evaluated. Human serum albumin may reduce adsorption and improve colloidal stability, but its source, quality, and concentration require control. It may also interfere with total protein measurements and particle-to-protein ratios. Trehalose may mitigate freeze- and aggregation-related damage, but the useful concentration is product- and formulation- dependent. Particle recovery alone is insufficient if cargo integrity or potency is not maintained.

Container development should be conducted in conjunction with formulation development because EVs can adsorb to glass, polymers, stoppers, tubing, and other contact materials. The risk may be greater at low concentrations, low fill volumes, or high surface-to-volume ratios. Studies should assess particle recovery, aggregation, extractables and leachables, closure compatibility, fill volume, headspace, storage orientation, and any administration device included in the presentation (International Conference on Harmonisation of Technical Requirements for Registration of Pharmaceuticals for Human Use, 1995; International Council for Harmonisation of Technical Requirements for Pharmaceuticals for Human Use, 2025b). Data generated in research-grade tubes should not be assumed to represent the performance of the intended clinical or commercial container. When alternative containers are used during early development, bridging studies should address relevant differences before those data are used to support the intended presentation or shelf life.

### 4.5 Lyophilized products

Lyophilization may reduce dependence on frozen storage and transport, but it creates a distinct product presentation with its own formulation, manufacturing process, container, and reconstitution requirements. Similar particle concentration and mean size before and after lyophilization do not, by themselves, establish stability.

Charoenviriyakul et al. reported that trehalose reduced aggregation and preserved selected protein, RNA, and cargo-related activities of B16BL6-derived exosomes for approximately four weeks at 25 °C (Charoenviriyakul et al., 2018). In contrast, lyophilization without a protective excipient resulted in marked aggregation. These findings support the feasibility of lyophilization under the tested conditions but do not justify a general room-temperature shelf-life claim.

Frank et al. used EV-associated beta-glucuronidase as a model functional cargo and reported the preservation of enzyme activity under selected freeze-drying conditions (Frank et al., 2018). Subsequently, Trenkenschuh et al. found that a formulation containing 5% sucrose, 0.02% poloxamer 188, and low-concentration phosphate buffer maintained particle size and concentration for six months at 40 °C, whereas beta-glucuronidase activity declined between one and six months (Trenkenschuh et al., 2022). The difference between physical and functional results shows the importance of including a potency-related endpoint in stability assessment.

Lyophilization performance depends on source, formulation, freezing conditions, cycle parameters, and reconstitution (Lyu et al., 2022; Susa et al., 2023). Cycle development should consider relevant thermal properties and critical product temperature. Freezing rate, primary and secondary drying conditions, residual moisture, cake structure, and reconstitution time should be controlled (Lee et al., 2025). Stability testing should include assessment of cake appearance, residual moisture, reconstitution time, particle recovery, size distribution and aggregation, product-specific markers, cargo integrity, free cargo where relevant, and potency. Furthermore, container closure integrity, diluent quality, and post-reconstitution stability should be evaluated.

Accelerated studies can support formulation and cycle development and help identify degradation pathways. However, room-temperature shelf-life claims should be based on long-term data generated from representative batches in the intended container. Particle concentration alone is insufficient to establish stability.

### 4.6 Risk-based stability-indicating panel

The four-domain framework presented in Section 3 can guide the selection of stability-indicating attributes. Table 3 presents an illustrative panel for a 24-month long-term study. It is not intended as a universal testing schedule or specification. Methods, time points, and acceptance criteria should reflect product presentation, formulation, mechanism of action, administration route, development stage, and known or plausible degradation pathways.

**Table 3:** Illustrative risk-based stability-indicating panel for extracellular vesicle therapeutic products.

Category	Attribute	Representative procedure	Suggested timing in a 24-month long-term study	Main interpretation
General physicochemical	Appearance and visible foreign matter	Qualified visual inspection	Each scheduled time point	Detects visible precipitation, aggregation, discoloration, or container-related change.
General physicochemical	pH	Qualified potentiometric procedure	Each time point for liquid products and after reconstitution where relevant	Useful when formulation degradation or concentration change may alter pH
General physicochemical	Osmolality	Freezing-point osmometry	Initial, annual, and final time points, or when water loss or concentration change is plausible.	Not necessarily stability in every sealed or frozen presentation
Particle attributes	Particle concentration or recovery	Qualified NTA, TRPS, or another product-specific procedure	Each scheduled time point	Detects loss during storage or handling but does not establish EV identity.
Particle attributes	Size distribution and aggregation	Qualified particle-sizing procedure with a selected complementary method	Primary procedure at each time point. Complementary procedure at selected time points or during investigation	Detects aggregation or changes in the particle population
Identity and composition	Reduced product-specific marker profile	Capillary immunoassay, ELISA, or qualified flow cytometry	Initial, intermediate, and final time points. More frequent testing when shown to be stability indicating	The stability panel may be narrower than the extended characterization panel.
Structural characterization	Membrane integrity or morphology	Qualified membrane-integrity procedure or cryogenic electron microscopy	Selected initial, intermediate, and final time points	Best suited for characterization or investigation unless a routine procedure is available.
Cargo	Active cargo amount and integrity, when applicable	Product-specific immunoassay, activity assay, qPCR, dPCR, RT-qPCR, or RT-dPCR	Each scheduled time point when cargo is critical to activity	Total cargo amount should be distinguished from integrity and EV association
Cargo and purity	Free cargo or soluble product-related material	Fractionation followed by a cargo-specific procedure	Selected time points based on leakage or degradation risk	An increase may indicate cargo release or product disruption.
Formulation	Critical excipient content, when applicable	Product-specific chromatographic or biochemical procedures.	Initial, annual, and final time points when excipient degradation is plausible.	Routine testing is not needed for every excipient.
Potency	Product-specific biological activity	Qualified cell-based, biochemical, or other functional procedure	Each scheduled time point	Potency is central when biological activity may change during storage.
Container and microbiological control	Container closure integrity or sterility	Validated container closure integrity procedure or compendial sterility test	Initial and final under the current ICH Q5C. Annual testing may be considered if the final consolidated Q1 adopts the current draft recommendation.	Draft and final regulatory expectations should be distinguished.
Lyophilized products	Cake appearance and reconstitution time	Visual inspection and timed reconstitution	Each scheduled time point	Evaluates presentation quality and clinical usability
Lyophilized products	Residual moisture	Karl Fischer titration or another qualified procedure	Initial, annual, and final time points, or more often when supported by development data.	Moisture can affect both physical and functional stability.

**Table notes** 1. The table presents an illustrative design for a product with a proposed 24-month shelf life. The selected attributes and testing frequency should be justified for the defined product. 2. Formal registration studies should use representative batches and the proposed container closure system. Earlier development programs may use a stage-appropriate batch strategy. 3. The stability-marker panel may be narrower than the extended characterization panel if the selected procedures remain sensitive to relevant product changes. 4. Host-cell proteins, residual DNA, endotoxins, and other process-related impurities are generally controlled at release. They should be repeated during stability only when a plausible time-dependent change has been identified. 5. Current ICH Q5C identifies the initial and final time points as the minimum for sterility testing or an appropriate container closure integrity procedure (International Conference on Harmonisation of Technical Requirements for Registration of Pharmaceuticals for Human Use, 1995). The Step 2 consolidated Q1 draft proposes at least annual testing for a shelf life longer than 12 months (International Council for Harmonisation of Technical Requirements for Pharmaceuticals for Human Use, 2025b). The draft recommendation should not be presented as a current final requirement. 6. For lyophilized products, cake appearance, residual moisture, reconstitution performance, and post-reconstitution stability should be evaluated. 7. Acceptance criteria or development-stage limits should be justified using analytical capability, development data, and experience with material used in nonclinical and clinical studies. 8. Accelerated, stress, freeze–thaw, shipping, and in-use studies should be conducted as separate supportive studies. 9. The panel was informed by published studies on EV storage, freeze–thaw exposure, formulation, and lyophilization (Ahmadian et al., 2024; Bosch et al., 2016; Charoenviriyakul et al., 2018; Frank et al., 2018; Gelibter et al., 2022; Gorgens et al., 2022; Levy et al., 2023; Lyu et al., 2022; Susa et al., 2023; Trenkenschuh et al., 2022).

For proposed shelf lives exceeding 12 months, a conventional schedule includes testing every three months during the first year and every six months during the second year (International Conference on Harmonisation of Technical Requirements for Registration of Pharmaceuticals for Human Use, 1995; International Council for Harmonisation of Technical Requirements for Pharmaceuticals for Human Use, 2025b). The 0-, 3-, 6-, 9-, 12-, 18-, and 24-month schedule in Table 3 follows this pattern. Schedules may be justified by shelf life, development stage, prior knowledge, or reduced protocol designs. Formal registration studies should use representative material in the proposed container-closure system.

The core panel should monitor appearance, product recovery, size distribution and aggregation, relevant compositional attributes, and potency. Additional tests should be included when they can detect plausible degradation pathways. Extended methods such as cryogenic electron microscopy, broad proteomics, or detailed subpopulation analysis may be reserved for selected time points or investigations unless they have a defined stability role.

Sterility and container-closure integrity testing follow the applicable final guideline. Current ICH Q5C identifies the initial and final time points as the minimum requirement (International Conference on Harmonisation of Technical Requirements for Registration of Pharmaceuticals for Human Use, 1995). The Step 2 Q1 draft proposes annual testing for products with a shelf life exceeding 12 months (International Council for Harmonisation of Technical Requirements for Pharmaceuticals for Human Use, 2025b). Because Q1 remains a draft, annual testing should be presented as a draft-based example rather than a current regulatory requirement. Endotoxins, residual host-cell proteins, residual DNA, and other process-related residuals are generally controlled at release and should be reassessed during stability only when a time-dependent change is plausible.

Accelerated, stress, freeze-thaw, shipping, and in-use studies should be presented separately from the long-term schedule. Each should use either the full panel or a justified subset selected according to the expected failure mode. These studies should demonstrate that the analytical procedures detect relevant changes and support formulation, packaging, and handling decisions (International Council for Harmonisation of Technical Requirements for Pharmaceuticals for Human Use, 2023a, International Council for Harmonisation of Technical Requirements for Pharmaceuticals for Human Use, 2023e, International Council for Harmonisation of Technical Requirements for Pharmaceuticals for Human Use, 2025b).

### 4.7 Three-axis framework for describing EV stability evidence

Published stability findings should not be extrapolated between EV products based solely on storage temperature. Meaningful interpretation requires information on the biological source, upstream context, product presentation, storage conditions, downstream processing, formulation, concentration, fill volume, container-closure system, freeze-thaw history, study duration, and analytical endpoints. Although these variables may interact, available evidence does not support a general predictive model. Therefore, the proposed framework is intended as a reporting and evidence-comparison tool rather than a shelf-life model.

We propose three top-level axes for organizing stability evidence: biological source, product state and storage, and downstream isolation or purification. Source categories include MSC-derived, immune cell-derived, tumor cell-derived, plant- or milk-derived, and other defined EV sources. Tissue origin, donor or cell-bank history, passage level, culture format, medium composition, and conditioning procedures should be reported when available. Engineering should be documented as a product-design feature rather than as a separate source category.

The product-state axis should capture both the presentation and any transitions during the study, such as frozen liquid products subjected to refrigerated post-thaw holding or lyophilized product followed by reconstitution. The downstream axis should document the sequence of isolation and purification steps rather than a single platform designation. Relevant approaches may include ultracentrifugation, size-exclusion chromatography, tangential flow filtration, precipitation, affinity capture, and combined processes. Additional reporting descriptors proposed in this review include formulation, concentration, fill volume, container-closure system, handling history, storage duration, and analytical endpoints.

External evidence is most relevant when the product and study contexts are closely aligned. Evidence generated under different conditions may still support excipient selection, stress-condition design, or risk assessment, but it should not replace long-term data generated using the product under review.

A complete stability claim should specify the source and upstream context, manufacturing and purification process, product presentation, formulation, concentration, fill volume, container, storage and handling conditions, study duration, and the attributes used to define stability. For example, a statement that an EV product remained stable for 24 months at −80 °C is incomplete unless it identifies the product, formulation, container, thawing conditions, and attributes that remained within predefined acceptance criteria. Potency should be included whenever biological activity forms part of the stability-indicating strategy.

Furthermore, the framework clarifies the limits of published prior knowledge. A study may support excipient selection or stress-condition design without being sufficiently comparable to support shelf-life conclusions. The analytical procedures used must also be considered because unchanged particle count or mean size does not establish preservation of identity, cargo integrity, or potency.

**Fig. 4:**
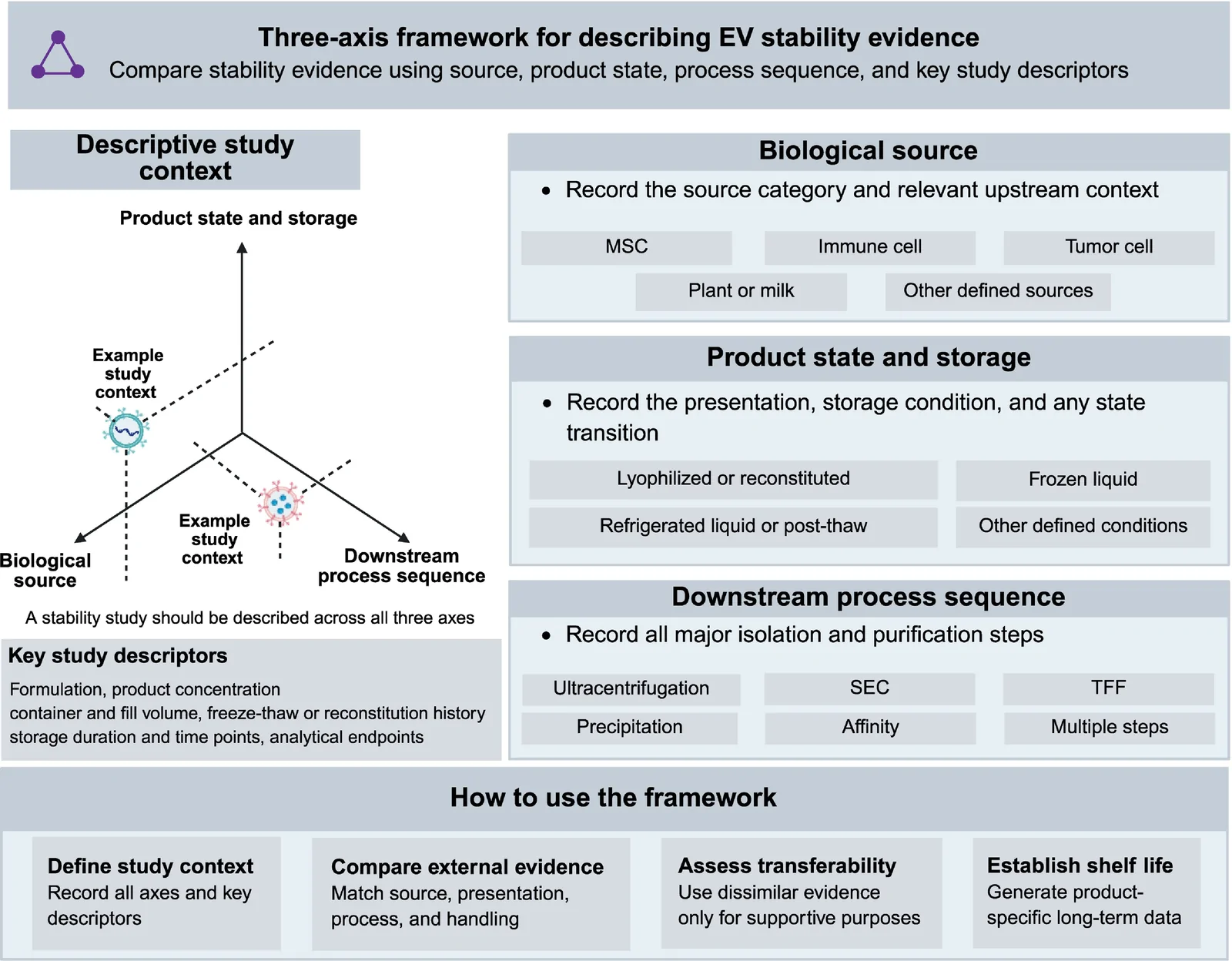
Three-axis framework for describing the context of stability evidence for extracellular vesicle therapeutic products. Stability evidence is organized by biological source, product state and storage, and downstream isolation or purification. The axes are descriptive and do not rank risk or assign numerical coordinates. Source context, process sequence, product-state transitions, formulation, concentration, fill volume, container, handling history, storage duration, and analytical endpoints should be reported. The framework assesses the relevance of external evidence and does not predict shelf life.

Table 3 and Fig. 4 serve different purposes. Table 3 presents candidate stability-indicating attributes and suggested testing frequencies. Fig. 4 defines the product and study context needed to interpret the results. Collectively, they support the selection of the fit-for-purpose analytical procedures discussed in Section 5.

## 5 Analytical control strategy for extracellular vesicle therapeutics

### 5.1 Fit-for-purpose analytical strategy

Analytical control should begin with the quality decision a result is intended to support. A procedure used to explore product composition during development may not be suitable for routine lot release, whereas a concise release procedure may be inadequate for process investigation, comparability, or characterization of a degradation pathway. Each procedure should therefore have a defined role in characterization, process development, in-process control, release, stability, or comparability before its performance requirements are established.

In this section, method class refers to a general measurement principle, whereas analytical procedure refers to the product-specific protocol used to generate a result. An analytical procedure includes sample preparation, instrument settings, calculations, data-processing rules, controls, and system-suitability criteria.

ICH Q14 uses the analytical target profile to define the intended purpose, measurand, and performance characteristics required to support a quality decision (International Council for Harmonisation of Technical Requirements for Pharmaceuticals for Human Use, 2023a). ICH Q2(R2) provides principles for validation according to intended use (International Council for Harmonisation of Technical Requirements for Pharmaceuticals for Human Use, 2023e). Relevant performance characteristics may include specificity or selectivity, accuracy, precision, reportable range, detection or quantitation limits, and robustness. Not all characteristics apply to every procedure, and the required studies should be selected before qualification or validation begins.

Suitability applies to a defined analytical procedure, rather than to a platform name. NTA, flow cytometry, qPCR, ELISA, and cell-based assay platforms can support release or stability testing only when the measurand, sample preparation, product matrix, concentration range, instrument settings, software controls, and data-processing rules are clearly defined. The resulting procedure must then demonstrate performance appropriate to its intended use. Familiarity with a platform cannot compensate for an ill-defined measurand or poorly controlled sample handling.

EV products require several procedures because particle number, size, marker profile, cargo, impurities, and biological activity describe different aspects of the product quality. The number and type of procedures should be guided by product risk assessment and the decision being supported. A larger analytical panel is not automatically more informative. Table 4 summarizes representative method classes and their potential roles within an analytical control strategy. These assignments are author proposed and do not indicate regulatory endorsement.

**Table 4:** Representative analytical method classes and possible roles in the control strategy for extracellular vesicle therapeutics.

Method class	Primary measurand	Main value	Key limitations and controls	Typical role in the control strategy
Nanoparticle tracking analysis (Arab et al., 2021; van der Pol et al., 2014; Vestad et al., 2017; Welsh et al., 2024)	Method-defined particle concentration and hydrodynamic size distribution	Rapid particle measurement with low sample-volume requirements	No EV specificity. Results depend on refractive index, instrument settings, dilution, viscosity, temperature, acquisition conditions, and software.	In-process monitoring and supportive release or stability testing after product-specific procedure development
Tunable or microfluidic resistive pulse sensing (Arab et al., 2021; van der Pol et al., 2014; Welsh et al., 2024)	Particle concentration and electrical size estimate within the selected pore range	Single-particle measurement independent of optical scattering	Pore selection, blockage, electrolyte composition, calibration, flow or pressure conditions, and size-dependent detection range	Independent particle assessment during method development, comparability, or investigation. Possible in-process or release use after qualification or validation
Dynamic light scattering (van der Pol et al., 2014; Welsh et al., 2024)	Ensemble hydrodynamic size and aggregation	Rapid and sensitive response to larger particles and aggregates	Intensity-weighted output is dominated by larger particles. It does not provide an EV-specific concentration.	Formulation screening and supportive aggregation monitoring during stability studies
Asymmetric flow field-flow fractionation with appropriate detectors (Welsh et al., 2024; Zhang et al., 2018)	Size-resolved particle populations, aggregates, and soluble co-isolates	Fraction-resolved analysis with optional light-scattering, ultraviolet, or refractive-index detection	Method complexity, membrane interaction, dilution, sample recovery, fraction collection, and detector-dependent quantitation	Extended characterization, comparability assessment, and investigation of aggregation or impurities
Cryogenic electron microscopy (Rikkert et al., 2019; Welsh et al., 2024; Yuana et al., 2013)	Vesicle morphology, bilayer structure, membrane integrity, and aggregation	Direct visualization with limited dehydration artifact	Low throughput, sampling bias, operator dependence, and image-selection requirements	Initial and extended characterization, comparability assessment, and selected stability investigations
High-sensitivity flow cytometry (Welsh et al., 2023; Welsh et al., 2020a)	Concentration and phenotype of marker-positive particles within a defined detection range	Single-particle marker analysis and marker co-detection	Detection threshold, swarm detection, antibody performance, fluorescence sensitivity, calibration, and reagent-lot effects	Identity and subpopulation characterization. Conditional routine use after rigorous procedure control
Single-particle interferometric reflectance imaging (Daaboul et al., 2016)	Immunocaptured particle count, size, and marker co-detection	Multiplexed phenotyping of captured particles	Capture bias, epitope accessibility, binding kinetics, chip design, and inability to measure the total population	Development characterization, comparability assessment, and supportive identity testing
Conventional Western blot (Welsh et al., 2024)	Presence and relative profile of selected proteins	Widely available and useful for supporting protein-marker characterization	Limited quantitative range, transfer variability, signal saturation, normalization problems, and operator dependence	Extended characterization and supportive identity assessment
Automated capillary immunoassay (International Council for Harmonisation of Technical Requirements for Pharmaceuticals for Human Use, 2023a; Welsh et al., 2024)	Quantitation of selected EV-associated, source-related, or cargo proteins	Greater automation and quantitative capability than conventional Western blotting	Antibody specificity, reference material, dilutional linearity, precision, and matrix effects require product-specific control	Potential release or stability testing for a defined protein attribute after qualification or validation
ELISA (International Conference on Harmonisation of Technical Requirements for Registration of Pharmaceuticals for Human Use, 1999; International Council for Harmonisation of Technical Requirements for Pharmaceuticals for Human Use, 2023a; Welsh et al., 2024)	Defined protein marker, cargo, host-cell protein, or soluble impurity	Quantitative and adaptable to routine testing	Does not distinguish soluble from EV-associated analyte without prior separation or capture	Release, stability, or impurity testing for defined analytes after qualification or validation
qPCR, dPCR, RT-qPCR, or RT-dPCR (Mateescu et al., 2017; Welsh et al., 2024)	Defined DNA or RNA cargo, residual nucleic acid, or vector-related sequence	High sensitivity and sequence specificity	Extraction recovery, reverse transcription for RNA, assay inhibition, normalization, sequence specificity, and free nucleic acid contamination	Release, stability, or impurity testing for defined nucleic acid attributes. Additional separation is needed when the EV association must be shown.
LC-MS proteomics (Subcommittee on Therapeutic Products Based on Extracellular Vesicles (EVs) Including Exosomes, 2023; Takakura et al., 2024; Welsh et al., 2024)	Broad protein composition, product-related variants, and impurities	High-content assessment of product composition	Complex sample preparation, normalization, limited routine standardization, and substantial data processing requirements	Extended characterization, process development, comparability assessment, and investigation of product change
Product-specific impurity procedure (International Conference on Harmonisation of Technical Requirements for Registration of Pharmaceuticals for Human Use, 1999; International Council for Harmonisation of Technical Requirements for Pharmaceuticals for Human Use, 2023a, International Council for Harmonisation of Technical Requirements for Pharmaceuticals for Human Use, 2023b, International Council for Harmonisation of Technical Requirements for Pharmaceuticals for Human Use, 2023e)	Defined host-cell protein, residual DNA, reagent, ligand, nuclease, excipient, or process-related impurity	Direct measurement of defined impurities or risks	A separate procedure may be needed for each impurity and product matrix.	In-process or release testing after qualification, validation, or compendial suitability assessment. Stability use when a time-dependent change is plausible
Cell-based functional bioassay (Gimona et al., 2021; International Conference on Harmonisation of Technical Requirements for Registration of Pharmaceuticals for Human Use, 1999; International Council for Harmonisation of Technical Requirements for Pharmaceuticals for Human Use, 2023a; U.S. Food and Drug Administration, 2011)	Product-relevant biological activity	Direct assessment of a response linked to the proposed mechanism of action	Biological variability, assay duration, cell-substrate control, calculation model, and reference-lot requirements	Primary potency procedure, stability procedure, comparability assessment, or component of a potency matrix
Reporter, biochemical activity, receptor-binding, or target-engagement procedure (Gimona et al., 2021; International Council for Harmonisation of Technical Requirements for Pharmaceuticals for Human Use, 2023a; U.S. Food and Drug Administration, 2011)	Defined mechanism-related activity	Quantitative output that may provide greater precision than a complex cell-based assay.	May not capture the full biological activity of a multicomponent product.	Supporting component of a potency matrix. Potential surrogate release procedure after a relationship with functional activity is demonstrated
Compendial or product-specific safety procedures (International Conference on Harmonisation of Technical Requirements for Registration of Pharmaceuticals for Human Use, 1999; International Council for Harmonisation of Technical Requirements for Pharmaceuticals for Human Use, 2023b; National Institute of Food and Drug Safety Evaluation, 2024)	Sterility, bacterial endotoxin, mycoplasma, adventitious agents, procoagulant activity, or relevant residuals	Established regulatory basis for some procedures and direct control of defined safety risks	Product-matrix interference and method suitability must be demonstrated. Some risks are controlled at the source, cell-bank, or process level rather than by final-product testing	Source, cell-bank, process, in-process, or release control according to product-specific risk

**Table notes** 1. Method class refers to a general measurement principle. Suitability for release, stability, or comparability applies to a defined analytical procedure with controlled sample preparation, instrument settings, calculations, data-processing rules, and controls. 2. The listed roles are illustrative and represent the authors' assessment. They do not indicate the regulatory endorsement of a method class. 3. Routine release or stability use requires product-specific procedure development and qualification, validation, verification, or compendial suitability assessment appropriate to the intended use and development stage. 4. A single-particle method does not necessarily provide EV specificity. Non-vesicular particles may be included unless the procedure incorporates a product-specific marker, capture step, or separation strategy. 5. Different particle platforms may detect different portions of the particle population. Results should not be assumed to be interchangeable, even when the platforms report the same nominal measurand. 6. Conventional Western blotting, cryogenic electron microscopy, broad proteomics, and other high-content methods are generally most suitable for characterization, comparability, or investigation unless additional evidence supports routine quantitative use. 7. An orthogonal procedure should measure the same or a closely related attribute using an independent principle. A procedure that measures a different attribute is complementary rather than orthogonal. 8. Commercial platform names should be omitted unless a platform-specific feature is essential to the discussion. 9. Selected references are illustrative rather than exhaustive.

### 5.2 Particle concentration, size distribution, and morphology

Particle concentration and size distribution can support process monitoring, dose assignment, aggregation assessment, and stability evaluation. However, they do not establish EV identity because the measured population may include protein aggregates, lipoproteins, formulation-derived particles, and other non-vesicular materials. Unless EV specificity has been demonstrated, results should therefore be reported as method-defined particle measurements rather than as total EV counts.

NTA estimates hydrodynamic size based on Brownian motion and derives particle concentration within a method-specific detection range. Results depend on refractive index, camera settings, threshold settings, dilution, diluent composition, viscosity, temperature, acquisition conditions, and software (Vestad et al., 2017). A controlled procedure should define these variables alongside replicate number, acceptable particle-count ranges, data-exclusion criteria, and system-suitability requirements. Sample concentration should be optimized to avoid sparse particle tracking and excessive particle overlap.

Resistive pulse sensing measures transient electrical changes as particles pass through a constriction and is independent of optical scattering. Performance depends on pore or cartridge selection, electrolyte composition, particle concentration, calibration materials, pressure or flow conditions, and pore blockage. Broad particle-size distributions may require multiple pore sizes or measurement ranges. NTA and resistive pulse sensing may yield different results because they detect different portions of the particle population. Similar considerations apply to flow cytometry and single-particle imaging (van der Pol et al., 2014). In a direct comparison of the same EV preparations, MRPS and NFCM yielded similar particle counts, whereas NTA reported concentrations approximately one order of magnitude lower for EVs, but not for synthetic particles. SP-IRIS, MRPS, and NFCM returned size profiles in which smaller particles predominated, whereas NTA detected a population with a modal diameter greater than 100 nm (Arab et al., 2021). In a separate study, two NanoSight NS500 instruments produced significantly different size and concentration results for the same EV sample aliquots under identical settings; instrument-specific optimization reduced these inter-instrument differences to nonsignificant levels for the EV samples (Vestad et al., 2017). These findings demonstrate that the analytical platform and operating settings can materially affect the reported particle concentration and size distribution. Results should therefore not be treated as interchangeable without side-by-side evaluation across representative lots, relevant concentration ranges, and challenging sample types.

DLS provides ensemble hydrodynamic size measurements and is highly sensitive to a small number of large particles. This characteristic makes it useful for detecting aggregation during formulation development or stability studies but less suitable for defining the primary size distribution of heterogeneous EV preparations. Therefore, it should not be used as a stand-alone procedure for identity assessment or particle quantification.

Asymmetric flow field-flow fractionation can separate particles and soluble materials according to hydrodynamic behavior before detection. Coupling with light scattering, ultraviolet absorbance, or refractive-index detection can provide information on aggregates, particle subpopulations, and co-isolated soluble materials (Zhang et al., 2018). Quantitative application requires evaluation of membrane interactions, sample recovery, dilution, fraction collection, detector response, and selective loss of subpopulations.

Cryogenic electron microscopy provides direct images of bilayer structures, morphology, aggregation, and membrane disruption (Rikkert et al., 2019; Yuana et al., 2013). It is useful for initial characterization, comparability, and investigation of aggregation or fusion. However, low throughput, sampling bias, operator dependence, and image-selection criteria limit its suitability for routine release testing. When qualitative conclusions are intended, fields of view, particle-selection criteria, and image-analysis rules should be predefined.

### 5.3 Identity and marker-based procedures

Identity testing should distinguish the intended product from unrelated particles and from EV preparations generated by unintended sources or manufacturing process. Common tetraspanins alone are insufficient. A product-specific identity strategy should incorporate relevant EV-associated markers, source-related or engineered features when available, and markers of unwanted cellular or extracellular materials (Welsh et al., 2024). Physical particle measurements and protein-marker procedures address different questions and should have defined roles.

High-sensitivity flow cytometry can quantify marker-positive particles and assess marker co-detection within a defined detection range. Results should be expressed as marker-positive events or particles above qualified scatter or fluorescence thresholds, not as total EV concentration. Analytical procedures should evaluate coincidence or swarm detection through sample dilution and include buffer, reagent, unstained, and biological controls. Detergent treatment can provide supportive evidence but should not serve as the sole demonstration of vesicular identity. Fluorescence calibration, antibody titration, reagent-lot control, and reporting of the lowest detectable signal are also important. MIFlowCyt-EV and the related compendium provide reporting guidance but do not constitute a validated release procedure (Welsh et al., 2023; Welsh et al., 2020a).

Single-particle interferometric reflectance imaging combines immunocapture with particle counting, size estimation, and marker detection (Daaboul et al., 2016). It can characterize capture-defined subpopulations, but results depend on antibody specificity, epitope accessibility, binding kinetics, chip design, and the analysis algorithm. Measured counts represent the captured subpopulation rather than the total particle population. These limitations should therefore be considered when interpreting identity and comparability data.

Conventional Western blotting remains useful for confirming selected protein markers during extended characterization studies. However, limited quantitative range, transfer variability, signal saturation, normalization challenges, and operator dependence make it less suitable for routine quantitative release testing. Automated capillary immunoassays may improve standardization and quantitative performance, but antibody specificity, dilutional linearity, reference materials, precision, and matrix effects still require product-specific control.

ELISA can quantify a defined protein marker, cargo, host-cell protein, or soluble impurity. However, a bulk measurement does not establish association of the analyte with EVs. When EV association is part of the identity claim, prior separation, immunocapture, or another appropriate procedure should be used to distinguish EV-associated analytes from soluble materials.

### 5.4 Cargo, purity, and process-related impurity assessment

A therapeutic cargo may contribute to identity, dose, potency, purity, and stability. The analytical strategy should distinguish among total amount, structural integrity, association with the EV fraction, location or accessibility when relevant, and unassociated material in the formulation. These distinctions are especially important for products loaded with proteins, RNA, DNA, or synthetic oligonucleotides.

Protein cargo may be measured using immunoassays, capillary immunoassays, activity assays, or targeted mass spectrometry. Defined DNA cargo and residual DNA may be measured by qPCR or dPCR, whereas RNA cargo requires RT-qPCR or RT-dPCR. Analytical procedures should evaluate extraction recovery, reverse-transcription efficiency for RNA, assay inhibition, reportable range, sequence specificity, normalization, and recovery in the final product matrix.

Detection of cargo in a bulk preparation does not establish that it is contained within or stably associated with EVs. Fractionation, immunocapture, or nuclease- and protease- protection studies performed with and without membrane disruption can provide information on cargo association and topology (Mateescu et al., 2017; Welsh et al., 2024). Appropriate controls are needed to account for incomplete digestion, detergent effects, and analyte recovery. When unassociated cargo may affect dose, potency, or safety, it should be measured separately.

RNA sequencing and global proteomics can reveal compositional differences among sources, processes, or lots. These approaches are most useful during product definition, process development, comparability, and investigation of product change. Routine release application is more challenging because variability may be introduced through sample preparation, normalization, data processing, and database selection. Once a relevant marker is identified, a targeted analytical procedure is generally more appropriate for routine control.

Purity assessment should focus on defined product- and process-related impurities. Relevant procedures may include host-cell protein assays, residual host-cell DNA procedures, assays for residual affinity ligands or nucleases, chromatographic measurement of excipients, and procedures for free cargo or soluble protein. Recovery, dilutional linearity, and matrix interference should be evaluated in the final formulation.

Total protein and the particle-to-protein ratio may support process comparisons within a defined manufacturing platform but are not universal measures of purity (Webber and Clayton, 2013). Total protein may include EV-associated proteins, soluble proteins, and protein excipients. The ratio is difficult to interpret in an albumin-containing formulation unless the excipient contribution is separated or otherwise accounted for (Gorgens et al., 2022). No single result provides a complete description of purity. Impurity-specific procedures should be combined with information on particle populations and soluble material, with recovery and selective loss documented for separation-based methods.

### 5.5 Potency procedures

The primary potency procedure should measure a biological activity relevant to the proposed mechanism of action or intended therapeutic effect. It should provide a quantitative result within a defined range and detect meaningful changes caused by manufacturing or storage (Gimona et al., 2021; International Conference on Harmonisation of Technical Requirements for Registration of Pharmaceuticals for Human Use, 1999). A dose-response relationship should be demonstrated when relevant. Depending on the product, the response may involve immunomodulation, target-cell killing, tissue repair, receptor activation, intracellular cargo delivery, enzyme activity, or another product-relevant function.

Cell-based procedures should not be excluded from release solely because they are slower or more variable than physicochemical procedures. They can support release and stability testing when the cell substrate, passage range, culture conditions, reference lot, positive and negative controls, dose-response model, calculation rules, and system-suitability criteria are controlled. Intermediate precision and dilutional parallelism are especially important for relative-potency assays.

A potency matrix should have a clear hierarchy. At least one procedure should measure the intended biological function. Supporting procedures may evaluate active cargo, receptor binding, cellular association, target engagement, or downstream biochemical responses. Several weakly related assays do not create a strong potency strategy. A surrogate procedure should support routine release only after its relationship with functional activity has been demonstrated across representative and intentionally altered lots.

Potency procedures used in stability studies should detect storage-related changes. Relevant stressed materials may include thermal, freeze-thaw, agitation, oxidation, light, or other product-specific conditions. Stress conditions should produce interpretable changes associated with plausible degradation pathways. A procedure that yields similar results for intact and functionally altered materials is not suitable for shelf-life assessment.

An exploratory mechanism-related assay does not necessarily need to become the final release procedure. The potency strategy may evolve as the mechanism of action, manufacturing process, and sources of variability become better understood. Changes in assay format, cell substrate, calculation model, or reference lot should be bridged when they could affect the interpretation of longitudinal data.

### 5.6 Safety-related procedures

Compendial or established safety procedures generally have a clearer regulatory foundation than EV-specific characterization procedures. However, sterility, bacterial endotoxin, mycoplasma, and related tests still require demonstration of suitability within the EV product matrix. Particles, protein excipients, surfactants, and other formulation components may introduce inhibition, enhancement, adsorption, or recovery effects. Dilution, spike recovery, interference testing, and matrix controls should be included as appropriate.

Viral safety cannot be reduced to one final-product procedure. It includes donor or source qualification, cell-bank testing, raw-material control, manufacturing controls, and testing at justified stages (International Council for Harmonisation of Technical Requirements for Pharmaceuticals for Human Use, 2023b). The analytical strategy should distinguish lot-release testing from controls established through cell-bank qualification, raw-material control, or process validation.

Additional procedures may be required to address product-specific risks, including procoagulant activity, residual vector sequences, unintended transgene products, immunogenic surface components, or tumor-associated materials. Each procedure should be placed where the risk can be controlled most effectively. Not every safety-related procedure belongs in the routine release panel.

The same fit-for-purpose principles apply to noncompendial safety procedures. The measurand, product matrix, sensitivity, controls, and decision limits should be clearly defined. For complex or multivariate procedures, data processing and model maintenance should be controlled throughout the analytical lifecycle (International Council for Harmonisation of Technical Requirements for Pharmaceuticals for Human Use, 2023a, International Council for Harmonisation of Technical Requirements for Pharmaceuticals for Human Use, 2023e).

### 5.7 Orthogonality, reference materials, and analytical lifecycle management

Orthogonal and complementary procedures serve different purposes. Orthogonal procedures measure the same or a closely related attribute using independent principles, whereas complementary procedures evaluate different aspects of the product (Simon Jr. et al., 2023). NTA and resistive pulse sensing may provide independent measurements of overlapping particle populations, but their detection characteristics differ. In contrast, a particle-counting procedure and a protein-marker procedure are complementary because they measure different attributes.

MISEV2023 supports the use of complementary evidence but does not prescribe a fixed number of procedures for therapeutic lot release (Welsh et al., 2024). Analytical depth should depend on attribute risk, specificity of the primary procedure, maturity of product knowledge, and the consequences of an incorrect result. A universal requirement for three orthogonal procedures is therefore not justified. An independent physical principle may be valuable during procedure development, comparability, or investigation, while routine release may rely on one well-controlled procedure once its performance and relationship to other procedures are understood.

Reference materials should be classified by purpose. Instrument calibrants verify properties such as size, concentration, optical response, or fluorescence. Procedural controls monitor sample preparation and assay performance. Internal product reference lots may support relative potency or compositional procedures, whereas external commutable materials may support comparisons across methods or laboratories. These categories are not interchangeable.

No single EV reference material is suitable for all physical, phenotypic, compositional, and functional procedures. Recombinant EV-like particles and commercial EV preparations can support specific applications, such as instrument control, procedural assessment, or intermethod comparison (Geeurickx et al., 2019; Poudel et al., 2025; Welsh et al., 2020b). The suitability depends on purpose, homogeneity, stability, assigned value, and commutability for the specific procedure. These materials should not be described as universal EV standards.

Procedures that rely on an internal reference lot require a plan for storage, monitoring, qualification, replacement, and bridging. Bridging may also be needed after instrument replacement, major software updates, critical reagent changes, or transfer to another laboratory. The extent should reflect the risk associated with the change and the role of the procedure (International Council for Harmonisation of Technical Requirements for Pharmaceuticals for Human Use, 2023a).

### 5.8 Method selection and placement in the EV control strategy

A practical method-selection process begins with the quality decision to be supported. Developers should define the attribute and measurand, establish the analytical target profile where appropriate, select a primary procedure, and decide whether additional procedures are needed. The procedure should then be assigned to characterization, in-process control, release, stability, or comparability before qualification or validation requirements are defined.

The release panel should be narrower than the full characterization package. It should contain procedures that are sufficiently specific, precise, robust, and transferable for routine quality decisions. More extensive procedures may be reserved for key development milestones, significant process changes, comparability studies, or investigations triggered by atypical results.

Table 4 should be interpreted alongside the illustrative stability panel in Table 3. Table 3 presents candidate attributes and suggested testing frequencies, whereas Table 4 summarizes method classes that may be used to measure those attributes. Analytical procedures used in stability studies should be shown to detect relevant product changes, especially for particle, marker, cargo, and potency measurements.

The overall package should address relevant identity, purity, potency, and safety-related quality questions without repeating the complete characterization program for every lot or stability time point. Procedure selection should reflect the source, manufacturing process, formulation, product design, mechanism of action, route, and development stage.

Emerging single-particle, spectroscopic, microfluidic, and multiomic technologies can provide valuable development information. Routine control use should depend on a defined measurand, suitable performance, controlled software and data processing, and transferability. Novelty alone does not establish regulatory value. The same principles apply to Section 6, where the study must define what a detected signal represents and whether the label or reporter remains associated with the administered product.

## 6 Nonclinical biodistribution assessment of extracellular vesicle therapeutics

### 6.1 Objectives, study questions, and regulatory principles

Nonclinical biodistribution studies should characterize the location, persistence, and apparent clearance of a defined product-associated entity at the administration site, in blood or other relevant biofluids, and within target and nontarget tissues. The results should support the interpretation of pharmacology and toxicology findings and may inform dose selection, route, dosing interval, and clinical monitoring. Study objectives should clearly define the measured entity and the development decision the data are intended to support.

Selected pharmacokinetic concepts can be informative, but plasma concentration alone cannot describe EV product disposition. After administration, membrane components, surface proteins, luminal cargo, and labels may dissociate from one another. Consequently, a detected signal may represent product-associated material, membrane fragments, released cargo, free label, or a label-derived catabolite. In this section, the measured entity refers to the product component or label generating the analytical signal. Interpretation depends on how specifically that signal reflects the administered product.

The PMDA Science Board report and MFDS guideline identify biodistribution as relevant to EV therapeutic development, but neither specifies a universal analytical procedure, tissue panel, or sampling schedule (National Institute of Food and Drug Safety Evaluation, 2024; Subcommittee on Therapeutic Products Based on Extracellular Vesicles (EVs) Including Exosomes, 2023). Although ICH S12 formally applies to gene therapy products, its general principles can inform EV studies with product-specific justification (International Council for Harmonisation of Technical Requirements for Pharmaceuticals for Human Use, 2023c). These principles include the use of a representative test article, administration *via* the intended clinical route where feasible, a biologically suitable animal model, risk-based selection of tissues and time points, and integration with pharmacology or toxicology studies when appropriate.

Study objectives should be defined before selecting the labeling strategy and analytical procedure. Depending on the development question, studies may evaluate early systemic exposure, target-site delivery, persistence in nontarget tissues, apparent clearance, repeat-dose accumulation, cellular association, or the effect of a manufacturing or product-design change. A single study need not address all of these questions. Study design should reflect the proposed mechanism of action, clinical route, dose and dose concentration, dosing schedule, formulation, source cell, and known or plausible safety risks.

### 6.2 Product and study factors affecting biodistribution

Observed distribution profiles are shaped by the product, labeling strategy, and study design. Product-related factors include source cell, manufacturing and purification, particle concentration, size distribution, aggregation, surface composition, formulation, cargo, and targeting or engineering features. Study-related factors include route and rate of administration, dose and dose concentration, dosing frequency, animal species, disease state, immune status, sex, age, and sampling schedule. Label chemistry, labeling efficiency, free label content, and the stability of product-label association may further influence the observed distribution profile.

The route and procedure of administration are major factors. Wiklander et al. demonstrated that EV source, route, and targeting modifications affected organ-level signals in mice (Wiklander et al., 2015). Consequently, findings obtained following intravenous administration should not be directly extrapolated to intramuscular, intranasal, inhaled, subcutaneous, or other locally administered products. Whenever feasible, the intended clinical route should be used, and the administration, device, volume, dose concentration, and delivery rate should reflect the planned clinical procedure.

Although the liver, spleen, and lung signals are frequently reported after systemic administration, this pattern is not universal. Apparent profiles depend on the detection procedure, source material, route, dose, dose concentration, animal model, and sampling time (Smyth et al., 2015; Wiklander et al., 2015). Lung signal may reflect tissue association, vascular retention, aggregation, rapid administration, or labeling-related change. Accordingly, a single early signal should not be interpreted as intrinsic tissue tropism.

Surface composition may also affect the profile. Modification of EV surface glycosylation altered lung and lymph-node signals in mice (Royo et al., 2019). Producer-cell culture, purification, formulation, or storage may change surface proteins, glycans, lipid organization, aggregation, and interactions with serum components. These factors should be considered in the biodistribution risk assessment for manufacturing comparability. *In vivo* studies are not necessarily required when analytical and functional comparability provide sufficient evidence that distribution is unlikely to change.

The test article should represent the intended clinical product in attributes that may affect disposition. The same source, manufacturing process, formulation, concentration range, storage condition, and handling procedure should be used where possible. If labeling requires additional processing, labeled and unlabeled preparations should be compared before administration. Relevant comparisons include particle recovery, size distribution, aggregation, product-specific identity or composition markers, cargo integrity, and potency. Label burden, labeling efficiency, and unassociated label should also be reported.

The animal model should permit evaluation of intended pharmacology and plausible off-target exposure. Healthy animals may be appropriate for initial studies, but disease models may be necessary when inflammation, altered vascular permeability, tissue injury, tumor burden, or immune activation are expected to affect disposition. Species-specific interactions between product components and host cells should also be considered. The rationale for species, sex, disease and immune status, dose, route, and administration procedure should be documented.

### 6.3 Labeling strategy and analytical qualification

The central analytical challenge is determining how closely the signal represents the defined measured entity. Label selection should therefore consider signal specificity, stability of product-label association, sensitivity, quantitative range, tissue background, and effects on product quality. Label burden should remain low enough to minimize changes in product behavior while still providing adequate sensitivity.

Lipophilic fluorescent dyes are simple to use and compatible with optical imaging, but they have important limitations. Such dyes can form non-vesicular particles, persist after incomplete purification, transfer between membranes, or remain detectable after disruption of the labeled product. Pužar Dominkuš et al. demonstrated that PKH26 labeling produced dye-containing nanoparticles that generated false-positive cellular association signals (Puzar Dominkus et al., 2018). A dye-only control should undergo the complete labeling and purification procedure. Removal of unbound dye and stability of product-associated fluorescence should be assessed in formulation buffer and relevant biological matrices. Results should be described as dye-associated signal unless continued product association has been demonstrated.

Genetically encoded reporters may strengthen the link between a signal and a defined EV-associated protein (Lai et al., 2014). Examples include luciferase or fluorescent proteins fused to CD63 or another membrane-associated protein. This approach requires modification of the producer cell and may alter composition, surface structure, particle production, or tissue distribution. Reporter-bearing preparations should therefore be compared with the intended therapeutic product. A comparison of fluorescent, bioluminescent, and radioactive tracers found that a CD63-linked NanoLuc reporter altered the apparent distribution profile (Lazaro-Ibanez et al., 2021). Reporter linkage alone does not demonstrate the presence of intact EVs following administration.

Covalent fluorescent labeling may reduce spontaneous membrane transfer but can modify accessible surface proteins or lipids. Labeling degree and reaction conditions should therefore be controlled. Particle recovery, identity, cargo integrity, and potency should be compared before and after labeling. Analytical procedures should distinguish product-associated fluorophores from free fluorophores and labeled fragments.

Radiolabeling with positron- or gamma-emitting tracers can provide sensitive whole-body imaging and quantitative tissue analysis (Hong et al., 2021; Khan et al., 2022; Lazaro-Ibanez et al., 2021). Interpretation depends on radiochemical purity, labeling efficiency, specific activity, radionuclide half-life, stability of product-label association, and the fate of released radionuclides or labeled catabolites. A radioactive signal does not establish intact EV presence. Label stability should therefore be evaluated in formulation buffer, plasma, and other relevant matrices before *in vivo* studies are conducted. Whenever feasible, free radionuclides or a relevant breakdown product should be included as a control.

A product-intrinsic marker may be useful when the product contains a unique cargo, nucleic acid sequence, enzyme, or protein that can be measured specifically. qPCR, dPCR, immunoassays, or activity-based procedures may be appropriate when host-tissue background is absent or sufficiently low. The analytical procedure should establish recovery, matrix interference, sensitivity, reportable range, and the relationship between the measured marker and the administered product. A cargo-derived signal may describe cargo distribution but does not necessarily establish the distribution of intact EV-associated material.

Labeling and detection procedures should be qualified before the definitive study. Qualification should establish labeling efficiency, consistency across independently labeled preparations, removal and quantification of unbound label, and stability of product-label association in formulation and relevant biological matrices. Predefined criteria should compare labeled and unlabeled preparations for particle recovery, size distribution, aggregation, product-specific markers, cargo integrity, and potency. If labeling introduces a meaningful change, the labeled preparation should be regarded as a surrogate test article rather than assumed to represent the clinical product.

Analytical performance should be evaluated in each relevant tissue matrix, or within a scientifically justified matrix group. Assessments should include sensitivity, precision, reportable range, recovery, matrix interference, signal decay, and tissue background. Appropriate controls may include vehicle, free label, a process-matched label-only preparation, unlabeled product, and blank tissue. Independent confirmation should be considered when label dissociation is likely, when findings support an important safety conclusion, when unexpected profiles are observed, or the primary procedure cannot distinguish product-associated material from released label. Confirmation may involve a second label, a product-intrinsic marker, tissue fractionation, or a spatially resolved method (International Council for Harmonisation of Technical Requirements for Pharmaceuticals for Human Use, 2023a, International Council for Harmonisation of Technical Requirements for Pharmaceuticals for Human Use, 2023e).

### 6.4 Modular framework for biodistribution assessment

**Fig. 5:**
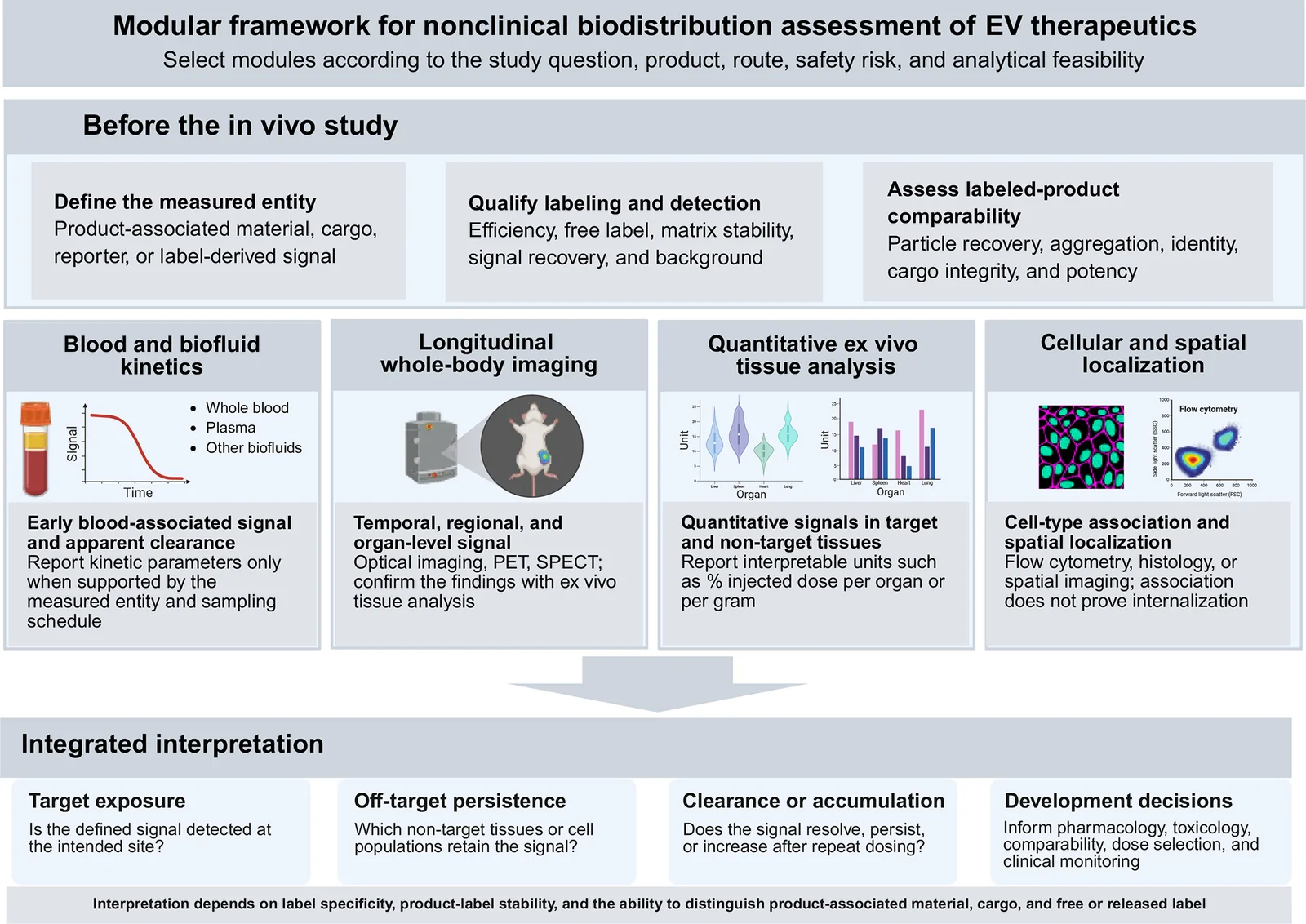
Modular framework for nonclinical biodistribution assessment of extracellular vesicle therapeutic products. Before the *in vivo* study, the measured entity should be defined, labeling and detection procedures qualified, and the labeled preparation compared with the unlabeled product. Four modules can then be selected according to the study question and product risk. Blood and biofluid sampling describes early signal kinetics and apparent clearance. Longitudinal imaging shows temporal regional and organ-level signal. Quantitative *ex vivo* analysis measures target and nontarget tissue signal. Flow cytometry, histology, and spatial methods identify associated cell populations and tissue regions. The modules are complementary rather than sequential. Interpretation depends on label specificity, product-label stability, and distinction among product-associated material, cargo, and free or released label.

We propose a modular framework that can include blood and biofluid kinetics, longitudinal whole-body imaging, quantitative *ex vivo* tissue analysis, and cellular or spatial localization. Each module addresses a distinct question and should be selected according to the product, route, mechanism of action, safety risks, and analytical feasibility. The modules are not intended to follow a fixed sequence, and not every product requires all four. Fig. 5 summarizes the framework.

#### 6.4.1 Blood and biofluid kinetics

Blood sampling can characterize early signal kinetics, systemic availability of the measured entity, and apparent clearance. Early sampling is particularly important after intravenous administration because rapid redistribution may occur (Lazaro-Ibanez et al., 2021; Wiklander et al., 2015). The sampling schedule should reflect pilot data, assay sensitivity, and the expected kinetics of both the product and label. For locally administered products, limited systemic sampling may be sufficient when justified.

Whole blood, plasma, serum, and cellular fractions provide different information. Product-associated materials may bind circulating cells, plasma proteins, or complement components; therefore, plasma alone may underestimate the blood cell-associated signals. Matrix selection should be justified and supported by recovery and stability data. Anticoagulant, processing time, centrifugation, and storage should also be controlled.

Concentration-time data may support Cmax, Tmax, area under the curve, and apparent terminal half-life when the procedure measures a defined product-associated entity and sampling captures the relevant phases. These parameters should not be presented as pharmacokinetics of intact EVs when only a label or cargo is measured. An apparent half-life should not be reported when the terminal phase is poorly defined or persistent label contributes materially.

Additional biofluids may be included to address specific pharmacological or safety questions. Examples include cerebrospinal fluid, bronchoalveolar lavage fluid, urine, bile, synovial fluid, and vitreous fluid. Matrix-specific recovery, dilution effects, background, and stability should be established before quantitative comparisons are made.

#### 6.4.2 Longitudinal whole-body imaging

Whole-body imaging enables repeated measurements in the same animal and provides a temporal overview of regional signal. Optical imaging can be highly sensitive in small animals but is affected by tissue depth, absorption, scattering, substrate delivery, and limited quantitative accuracy. PET and SPECT generally provide better tissue penetration and quantitative capability, although interpretation still depends on radiolabel stability and the fate of released radioactivity. None of these methods, however, demonstrates intact EV distribution by itself.

When organ-level quantitation is a primary objective, imaging should be complemented by *ex vivo* tissue analysis at selected time points. Signal overlap, limited spatial resolution, and tissue-dependent attenuation can obscure adjacent organs. Regions of interest, background subtraction, calibration, and normalization rules should be defined before analysis.

Where feasible, the field of view should include the administration site and regional drainage pathways. Local persistence, leakage, and lymphatic drainage may be particularly important after intramuscular, subcutaneous, intradermal, inhaled, intranasal, or other locally administered routes. Time points should capture both early movement and later persistence or clearance.

#### 6.4.3 Quantitative *ex vivo* tissue analysis

*ex vivo* tissue analysis can compare tissues quantitatively when recovery and calibration are established. The panel should include the administration site, target tissue, major clearance organs, and tissues associated with plausible off-target risks. An initial panel may include the liver, spleen, lungs, kidneys, heart, brain, gastrointestinal tract, draining lymph nodes, and blood. Final selection should reflect administration route, disease model, sex, product design, pharmacology, toxicity findings, and prior biodistribution data.

Perfusion should be considered when residual blood may contribute materially to the measured signal. Although perfusion can reduce intravascular background, it may also remove loosely associated material. Therefore, the procedure and its rationale should be predefined and reported. Tissue collection order, weighing, processing, homogenization, storage, and analytical recovery should be standardized.

Results should be reported using quantitative and interpretable units, such as percentage of injected dose per organ, percentage of injected dose per gram, amount of a product-associated marker per gram, or another qualified concentration measure. When possible, normalization should be based on the amount actually administered. Raw fluorescence intensity or image area alone is insufficient without calibration and tissue-specific correction.

The sampling schedule should include early time points to characterize initial distribution and later time points to assess persistence or apparent clearance. Study duration should reflect both the expected residence of the measured entity and the persistence of the label or reporter. Repeat-dose studies should assess accumulation or altered distribution. Persistent label accumulation should not be interpreted as accumulation of intact product without additional evidence.

#### 6.4.4 Cellular and spatial localization

An organ-level signal does not identify the associated cell population or establish whether internalization has occurred. Cellular and spatial analyses are therefore particularly informative when the proposed mechanism involves interaction with a defined target-cell population, delivery of a functional cargo to that population, or a safety concern arising from association with nontarget cells. Intracellular delivery should not be treated as a general prerequisite for EV activity; depending on the product and mechanism of action, biological effects may arise through cell-surface interactions, extracellular actions, or delivery of internalized cargo. The required level of cellular or subcellular localization evidence should therefore be matched to the proposed mechanism of action and the specific pharmacological or safety question.

Flow cytometry of tissue-derived cell suspensions can quantify signal-associated populations and relate signals to phenotypic markers. However, tissue dissociation may alter membrane-associated signals, lose fragile cells, or transfer extracellular labels. Results should be described as cellular association unless internalization has been demonstrated. Processing controls, recovery assessment, and validation of dissociation are required.

Immunohistochemistry, immunofluorescence, *in situ* hybridization, autoradiography, and spatial imaging preserve tissue context. Co-detection of a product-derived signal and a cell marker demonstrates spatial association but does not establish intracellular delivery. Evidence of internalization may require higher-resolution imaging, extracellular-signal quenching, three-dimensional analysis, subcellular fractionation, or demonstration of a functional cargo response.

Cellular analysis should focus on product-relevant target and plausible off-target populations. Tissue selection should be guided by organ-level signal, pharmacological relevance, toxicology findings, and cell-specific safety questions.

### 6.5 Interpretation, reporting, and comparability

Biodistribution findings should be interpreted alongside pharmacology, toxicology, and product-quality data. A high organ signal does not establish therapeutic delivery, target engagement, or activity. Conversely, a low signal does not exclude an effect when a potent cargo reaches a small target-cell population. Biodistribution, cellular association, internalization, target engagement, and pharmacodynamic activity are related but distinct observations.

Study reports should describe the EV source, producer-cell and cell-bank context, manufacturing and purification, formulation, storage and thawing history, administered dose, dose unit, dose concentration, route, device, administration volume, and rate. They should also describe the animal model, sex, disease and immune status, labeling procedure, label burden, labeling efficiency, removal of unbound label, product-label stability, analytical recovery and sensitivity, tissue panel, sampling schedule, quantitative units, and normalization.

To the extent supported by the procedure, reports should distinguish signals associated with product material, therapeutic cargo, membrane components, reporter, and free or released label. When these entities cannot be distinguished, the limitation should be stated. Terms such as “EV accumulation”, “EV clearance”, or “EV uptake” should not be used when only a nonspecific dye or released reporter is measured. More accurate terms include dye-associated, cargo-derived, or reporter-associated signals.

The need for a new assessment should be evaluated when manufacturing or clinical changes could alter disposition. Relevant changes include a new source cell or cell bank, major purification change, altered size or aggregation, a new formulation, targeting ligand, modified surface glycosylation, changes in cargo loading, new route, substantial increases in dose or concentration, or a different schedule. A new *in vivo* study is not automatically required. Analytical, functional, and prior *in vivo* evidence may support bridging when comparability has been demonstrated and distribution is unlikely to change.

Animal data do not directly predict human tissue exposure because translation is limited by species, disease model, immune status, administration conditions, and label behavior. They can still identify target and nontarget tissues, support toxicology interpretation, guide clinical monitoring, and reveal product attributes that affect distribution. Common reporting that separates product characteristics, label behavior, organ-level signal, and cellular association would improve cross-study comparisons. Section 7 considers this need a priority for regulatory convergence.

## 7 Priorities for regulatory convergence in extracellular vesicle therapeutics

Across jurisdictions, the primary opportunity lies in technical convergence rather than uniform legal classification. EV products differ in source, engineering, cargo, manufacturing process, formulation, dose unit, route of administration, and mechanism of action. Therefore, convergence should not imply a common assay panel or uniform numerical specifications. Near-term efforts should focus on shared technical product descriptions, transparent CQA and control-strategy decisions, comparable measurement practices, and consistent reporting of stability and biodistribution studies. Table 5 distinguishes actions that can be implemented within individual programs from those requiring broader scientific or regulatory coordination.

**Table 5:** Proposed priorities and staged actions for regulatory convergence in extracellular vesicle therapeutic development.

Priority area	Near-term action	Evidence needed for broader convergence	Possible next step
Common technical product description	Use a consistent description of source tissue and cell type, donor or cell-bank strategy, cell state, engineering, cargo, culture, harvest, purification, formulation, container, dose unit, route, and proposed mechanism of action. Summarize the evidence linking the EV product as a whole and, where supported, identified components or features to the intended activity. For complex products, acknowledge multicomponent or pleiotropic activity, distinguish experimentally established contributors from proposed or incompletely defined mechanisms, and do not presume that the overall activity can be assigned to a single component.	Cross-jurisdiction case studies showing which product features affect classification and development expectations (Committee for Advanced Therapies, 2026; European Commission, 2009; European Medicines Agency, 2018, European Medicines Agency, 2025, European Medicines Agency, 2026; European Parliament and Council of the European Union, 2007; ICH Cell and Gene Therapy Discussion Group, 2025; Kuribayashi, 2025)	Consistent sponsor briefing packages, parallel or sequential scientific advice, and publication of product-classification case studies
CQA and control strategy	Distinguish candidate attributes from product-specific CQAs. Document the quality risk, supporting evidence, analytical procedure, control location, and acceptance criterion or development-stage limit for each selected CQA.	Evidence linking attribute variability to product function, safety, process performance, and manufacturing change (International Conference on Harmonisation of Technical Requirements for Registration of Pharmaceuticals for Human Use, 1999, International Conference on Harmonisation of Technical Requirements for Registration of Pharmaceuticals for Human Use, 2004, International Conference on Harmonisation of Technical Requirements for Registration of Pharmaceuticals for Human use, 2008, International Conference on Harmonisation of Technical Requirements for Registration of Pharmaceuticals for Human Use, 2009, International Conference on Harmonisation of Technical Requirements for Registration of Pharmaceuticals for Human Use, 2012; International Council for Harmonisation of Technical Requirements for Pharmaceuticals for Human Use, 2023d)	Regional points-to-consider documents, cross-agency case studies, and later international work if the topic becomes sufficiently mature.
Measurement comparability and reference strategy	Define the measurand and complete the analytical procedure. Use instrument calibrants, procedural controls, and internal product reference lots according to their assigned purposes.	Evidence of homogeneity, stability, assigned value, measurement uncertainty, commutability, and interlaboratory performance (Geeurickx et al., 2019; International Council for Harmonisation of Technical Requirements for Pharmaceuticals for Human Use, 2023a, International Council for Harmonisation of Technical Requirements for Pharmaceuticals for Human Use, 2023e; Poudel et al., 2025; Simon Jr. et al., 2023; Welsh et al., 2020b)	Collaborative studies involving metrology institutes, pharmacopeias, scientific societies, regulatory laboratories, and product developers.
Potency and manufacturing comparability	Use a functional procedure to anchor the potency strategy when the mechanism of action is complex or partly defined. Define the role of supporting assays, reference lots, and altered product controls. Establish a prospective comparability plan.	Procedure performance, dose-response behavior where relevant, discrimination of stressed or process-altered lots, stability-indicating capability, and relationship to product function (Gimona et al., 2021; ICH Cell and Gene Therapy Discussion Group, 2025; International Conference on Harmonisation of Technical Requirements for Registration of Pharmaceuticals for Human Use, 2004; U.S. Food and Drug Administration, 2011)	Multi-agency workshops, product case studies, and points-to-consider documents. A formal guideline or annex may be considered when the evidence base is mature.
Stability	Generate long-term data using the intended formulation and container closure system. Use a risk-based stability-indicating panel and separate accelerated, stress, freeze-thaw, shipping, and in-use studies. Report the full product and study context.	Cross-product evidence on degradation pathways, formulation and container effects, product-state transitions, and the sensitivity and transferability of stability-indicating procedures (ICH Q1 Expert Working Group, 2026; International Conference on Harmonisation of Technical Requirements for Registration of Pharmaceuticals for Human Use, 1995; International Council for Harmonisation of Technical Requirements for Pharmaceuticals for Human Use, 2025b)	Common reporting templates, regional guidance, and compendial method development. Formal international work may follow when sufficient evidence is available.
Biodistribution	Define the measured entity. Qualify labeling and detection procedures. Compare labeled and unlabeled preparations. Select study modules, models, tissues, and time points according to product risk and study objectives.	Concordance across labels or product-intrinsic markers, relationships with pharmacology and toxicology, model relevance, quantitative recovery, and interlaboratory performance (International Council for Harmonisation of Technical Requirements for Pharmaceuticals for Human Use, 2023c; National Institute of Food and Drug Safety Evaluation, 2024; Subcommittee on Therapeutic Products Based on Extracellular Vesicles (EVs) Including Exosomes, 2023)	Common reporting templates, agency points-to-consider documents, and multi-agency scientific workshops before consideration of formal harmonisation

**Table notes** 1. The actions in this table were proposed by the authors. They are not current regulatory requirements, agency commitments, or formally agreed harmonisation plans. 2. Near-term actions can be implemented within individual development programs without agreement on a common legal classification. 3. Possible next steps require different levels of scientific maturity, interlaboratory evidence, and regulatory coordination. 4. The table does not imply that all EV products should use the same assays, acceptance criteria, reference materials, stability schedules, biodistribution modules, or development pathways. 5. Future ICH work on ATMPs may be relevant to some EV products, but its applicability will depend on the product, its legal classification, and the final scope of the relevant ICH work.

### 7.1 Common technical product description and dossier structure

Legal classifications are unlikely to become identical in the near term because they are tied to regional statutes and regulatory structures. The 2025 ICH Cell and Gene Therapy Discussion Group report also notes that nomenclature and classification for advanced therapies are often defined by regional law and may remain outside the scope of ICH harmonisation (ICH Cell and Gene Therapy Discussion Group, 2025). Therefore, a practical first step for EV therapeutics is a common technical product description rather than a common legal category.

This description should identify the source tissue and cell type, donor or cell-bank strategy, relevant cell-state attributes, engineering steps, active or loaded cargo, culture conditions, harvest method, purification process, formulation, container closure system, dose unit, route of administration, and proposed mechanism of action. It should also summarize the available evidence linking the EV product as a whole to its intended biological activity and, where supported, the contributions of identified cargo, surface features, or other product attributes. For native or otherwise complex EV products, multicomponent and pleiotropic activity may be an inherent characteristic of the drug product. Attribution of the overall biological effect to a single component should therefore not be presumed or required as a general principle. Where one or more contributors are proposed, the strength and limitations of the supporting evidence should be stated, and experimentally established contributors should be distinguished from proposed or incompletely defined mechanisms.

A common description would not replace jurisdiction-specific classification. Rather, it would provide a consistent technical basis for regulatory evaluation and reduce differences in the use of terms such as native EV, engineered EV, cargo-loaded EV, exosome, and EV-based delivery system across submissions.

MISEV2023 provides a scientific basis for EV terminology, sample description, and characterization (Welsh et al., 2024), but it does not establish medicinal-product specifications. A release specification requires a defined attribute, an analytical procedure suitable for its intended use, an acceptance criterion, and a scientific rationale linking the result to product quality (International Conference on Harmonisation of Technical Requirements for Registration of Pharmaceuticals for Human Use, 1999; International Council for Harmonisation of Technical Requirements for Pharmaceuticals for Human Use, 2023a). The depth of evidence and control should reflect product complexity, uncertainty, and potential patient risk (International Council for Harmonisation of Technical Requirements for Pharmaceuticals for Human Use, 2023d).

### 7.2 Transparent CQA and control-strategy decisions

The framework presented in Fig. 3 and Table 2 can support a common approach to CQA decisions. Candidate attributes should not be treated as CQAs until their criticality has been assessed for the defined product. For each product-specific CQA, the dossier should describe the relevant quality risk, the evidence linking attribute variability to safety or efficacy, the analytical procedure, and the point of control within the product lifecycle. An acceptance criterion or development-stage limit should be provided when appropriate.

Attributes not designated as CQAs may still support extended characterization, process understanding, comparability, stability investigations, or method development. The rationale for including or excluding an attribute from routine control should be stated. CQA selection and control placement should be reviewed as manufacturing, analytical, nonclinical, and clinical knowledge develops (International Conference on Harmonisation of Technical Requirements for Registration of Pharmaceuticals for Human Use, 2004, International Conference on Harmonisation of Technical Requirements for Registration of Pharmaceuticals for Human use, 2008, International Conference on Harmonisation of Technical Requirements for Registration of Pharmaceuticals for Human Use, 2009; International Council for Harmonisation of Technical Requirements for Pharmaceuticals for Human Use, 2023d).

### 7.3 Measurement comparability and reference strategy

Measurement comparability is a practical target for early convergence. Results from the same platform can differ because of sample preparation, calibration, instrument settings, software, reagent performance, and data-processing rules. Consequently, a platform name alone is insufficient for comparison across laboratories or development programs. The measurand and the complete analytical procedure should be described.

A reference strategy should distinguish instrument calibrants, procedural controls, internal product reference lots, and external commutable materials. Instrument calibrants verify responses for size, concentration, optical signal, or fluorescence. Procedural controls monitor extraction, labeling, staining, and sample recovery. Internal product reference lots can support relative potency or compositional procedures. External commutable materials can support comparison across laboratories or methods. These materials are not interchangeable.

Synthetic particles, recombinant EV-like particles, and EV preparations derived from defined cell sources can support specific analytical purposes (Geeurickx et al., 2019; Poudel et al., 2025; Welsh et al., 2020b). However, no single material reproduces all relevant physical, phenotypic, compositional, and functional properties of an EV therapeutic. A material suitable for particle-count calibration may not be suitable for flow cytometry, cargo analysis, or potency testing.

Before an external material is used for interlaboratory comparison, its intended use, identity, homogeneity, stability, assigned value, measurement uncertainty, and commutability should be established. Submissions should also describe the qualification performed, storage and monitoring plans, replacement strategy, and known limitations. Although external materials can improve measurement comparability, they do not replace an internal product reference when a procedure depends on product-specific composition or biological activity.

### 7.4 Potency and manufacturing comparability

Potency represents a suitable area for early technical convergence because common principles can be agreed without requiring identical assays across every product. The strategy should measure activity relevant to the proposed mechanism of action, provide a quantitative result over an appropriate range, show precision suitable for its intended use, and detect meaningful changes caused by manufacturing or storage (Gimona et al., 2021; U.S. Food and Drug Administration, 2011).

A potency matrix is not simply a collection of assays. It should follow a clear hierarchy. At least one procedure should measure a biological function relevant to the intended activity. Supporting procedures may measure active cargo, receptor binding, enzyme activity, cellular association, intracellular delivery, target engagement, or a downstream biochemical response. The role of each result in release, stability, comparability, or process control should be defined.

For products with complex or partly defined mechanisms of action, we propose that a functional procedure should anchor the potency strategy. Supporting procedures can improve precision, support process monitoring, and help explain changes in activity. A surrogate procedure should support routine release only after its relationship with functional activity has been demonstrated across representative and intentionally altered lots.

Furthermore, potency is important in manufacturing comparability. ICH Q5E provides the general framework for assessing whether a manufacturing change could affect product quality, safety, or efficacy (International Conference on Harmonisation of Technical Requirements for Registration of Pharmaceuticals for Human Use, 2004). The 2025 ICH Cell and Gene Therapy Discussion Group recommended a broader risk-based comparability approach for complex advanced therapies based on the totality of evidence (ICH Cell and Gene Therapy Discussion Group, 2025). Although direct applicability depends on the product and its regulatory classification, this reasoning may inform EV development.

A prospective comparability plan should identify the product and process attributes most likely to change after modification of the source cell, culture process, purification method, formulation, scale, analytical procedure, or manufacturing site. It should define the procedures used to detect those changes and the evidence required to support comparability. Relevant stressed materials and controlled process variants can help assess procedure sensitivity. Convergence should focus on the information needed to justify the potency strategy, while assay formats, procedures, and acceptance criteria remain product-specific.

### 7.5 Common reporting for stability and biodistribution

Stability and biodistribution address different questions, but both are difficult to interpret when product and study conditions are incompletely reported. As of June 2026, the consolidated ICH Q1 guideline remained in draft stage and should not be cited as final guidance (ICH Q1 Expert Working Group, 2026; International Council for Harmonisation of Technical Requirements for Pharmaceuticals for Human Use, 2025b). ICH S12 provides harmonized biodistribution principles for gene therapy products (International Council for Harmonisation of Technical Requirements for Pharmaceuticals for Human Use, 2023c). Neither document provides EV-specific study designs, although both can inform product-specific planning.

A stability report should identify the biological source and upstream context, manufacturing and purification sequence, product presentation, formulation, concentration, fill volume, container closure system, storage condition, freeze-thaw or reconstitution history, study duration, time points, batches, analytical procedures, and attributes used to define stability. Long-term studies should be distinguished from accelerated, stress, shipping, freeze-thaw, and in-use studies. Table 3 presents an illustrative risk-based panel, and Fig. 4 describes the product and study context needed to assess the relevance of stability evidence. Neither should be interpreted as assigning or predicting shelf life.

A biodistribution report should define the measured entity and state whether the result represents product-associated material, therapeutic cargo, a membrane component, a reporter, a fluorophore, a radionuclide, or a label-derived product. It should also describe label burden, labeling efficiency, removal of free label, stability of product-label association, route, administration volume and rate, dose unit, dose concentration, animal model, tissue panel, sampling schedule, analytical recovery, quantitative units, and controls. Fig. 5 should be interpreted as a modular framework rather than a required sequence.

An EV-specific reporting template or regional points-to-consider document could provide a practical near-term step toward convergence while preserving product-specific procedures and study designs. Multi-agency or formal ICH activities should be considered only after sufficient product-level, interlaboratory, and regulatory experience has accumulated.

### 7.6 A staged route to regulatory convergence

Progress can occur at three levels: individual product development, scientific and technical collaboration, and formal regulatory coordination. These activities can proceed in parallel but require different evidence bases and decision-making processes.

At the product level, sponsors should use a consistent technical core across regional submissions and identify differences in classification, potency, stability, comparability, and nonclinical expectations early in development. Scientific advice should be sought before pivotal manufacturing strategies or nonclinical programs are finalized. For eligible products developed in both the United States and the European Union, FDA-EMA Parallel Scientific Advice may support concurrent discussion of major scientific questions (European Medicines Agency; U.S. Food and Drug Administration, 2021). This procedure can identify areas of agreement and disagreement but does not guarantee identical agency conclusions.

At the scientific and technical levels, priorities include interlaboratory procedure studies, qualification of reference materials, publication of complete performance data, and development of common reporting templates. This work should involve academic laboratories, product developers, contract testing laboratories, metrology institutes, pharmacopeias, scientific societies, and regulatory laboratories. The MFDS guideline and PMDA Science Board report provide useful source documents because they identify EV-specific development questions while retaining product-specific flexibility (National Institute of Food and Drug Safety Evaluation, 2024; Subcommittee on Therapeutic Products Based on Extracellular Vesicles (EVs) Including Exosomes, 2023).

At the international level, a staged process is more realistic than the immediate development of a detailed EV guideline. An ICH reflection paper can frame a potential area for harmonisation and support later discussion of new topic proposals, guideline revisions, annexes, discussion groups, or training materials (International Council for Harmonisation of Technical Requirements for Pharmaceuticals for Human Use, 2025a). It does not, by itself, establish a guideline-development program. The 2025 ICH Cell and Gene Therapy Discussion Group report provides an example of staged scoping through targeted guidelines, annexes, and training activities rather than a single comprehensive document (ICH Cell and Gene Therapy Discussion Group, 2025).

Early international discussions on EV therapeutics should focus on technical product description, CQA and control-strategy decisions, analytical comparability, potency, manufacturing comparability, and reporting of stability and biodistribution studies. Differences in legal classification should not prevent progress on these technical areas.

The objective is not identical legal classification or a common assay panel. Rather, it is to ensure that similar scientific questions are addressed using evidence that can be understood and compared across jurisdictions. Differences in regulatory conclusions should then be traceable to the product, the evidence, and the applicable regional framework. Table 5 summarizes practical steps toward this objective.

## 8 Conclusion

EV therapeutics cannot be controlled through a universal marker panel, particle count, or platform label. Their composition and activity are shaped by biological source, manufacturing, formulation, storage, and engineering. Therefore, development should begin with a clear product definition and a control strategy that links these factors to quality risks.

The four-domain framework organizes candidate attributes into identity, purity, potency, and safety-related quality. It supports product-specific CQA selection rather than treating every measurable attribute as critical. Quantity remains a cross-cutting dose or normalization variable, whereas stability is assessed as a time-dependent change across the four domains. Additionally, safety control extends beyond final-product testing to source qualification, cell banks, raw materials, manufacturing controls, and nonclinical studies. Accordingly, the release specification should remain narrower than the full characterization package.

Stability and analytical control require the same product-specific logic. Shelf life should be supported by long-term data generated using the intended formulation, concentration, container, and handling conditions. Published findings are transferable only when product and study contexts are sufficiently comparable. The three-axis framework describes those contexts but does not predict shelf life. Analytical suitability belongs to a defined procedure and quality decision, not to a platform name. For products with a complex or partly defined mechanism of action, a functional procedure should anchor the potency strategy.

Biodistribution studies should define the measured entity before organ-level or cellular signals are interpreted. Labeling and detection procedures should be qualified, and labeled preparations should be compared with the intended product. Blood and biofluid kinetics, longitudinal imaging, quantitative tissue analysis, and cellular or spatial localization should be selected as complementary modules. Signal persistence should not be described as EV persistence unless continued association with the product has been demonstrated.

Legal classification will likely remain region-specific. Near-term progress can be achieved through common technical product descriptions, transparent CQA and control-strategy decisions, comparable measurement practices, and consistent reporting of stability and biodistribution studies. Collectively these measures would allow EV heterogeneity to be managed through product knowledge and risk-based control. The proposed frameworks should now be evaluated across products, laboratories, and development stages.

## CRediT authorship contribution statement

**Hyunjun Moon:** Writing – review & editing, Writing – original draft, Investigation, Conceptualization. **Suk Han Jung:** Writing – review & editing, Writing – original draft, Visualization, Investigation. **Sungjoon Bae:** Writing – original draft, Visualization. **Gyuri Bae:** Visualization, Investigation. **Areum Lee:** Visualization, Investigation. **Jin Hee Na:** Writing – review & editing, Investigation. **Jong-Ho Kim:** Writing – review & editing, Investigation. **Jeong Uk Choi:** Writing – review & editing, Supervision, Funding acquisition, Conceptualization. **Sangmin Lee:** Writing – review & editing, Supervision, Project administration, Conceptualization.

## Declaration of competing interest

The authors declare that they have no known competing financial interests or personal relationships that could have influenced the work reported in this paper.

## Data Availability

No new data were generated or analyzed in this review article.

## References

[bb0005] Ahmadian S., Jafari N., Tamadon A., Ghaffarzadeh A., Rahbarghazi R., Mahdipour M. (2024). Different storage and freezing protocols for extracellular vesicles: a systematic review. Stem Cell Res. Ther..

[bb0010] Almeria C., Weiss R., Roy M., Tripisciano C., Kasper C., Weber V., Egger D. (2019). Hypoxia conditioned mesenchymal stem cell-derived extracellular vesicles induce increased vascular tube formation in vitro. Front. Bioeng. Biotechnol..

[bb0015] Arab T., Mallick E.R., Huang Y., Dong L., Liao Z., Zhao Z., Gololobova O., Smith B., Haughey N.J., Pienta K.J., Slusher B.S., Tarwater P.M., Tosar J.P., Zivkovic A.M., Vreeland W.N., Paulaitis M.E., Witwer K.W. (2021). Characterization of extracellular vesicles and synthetic nanoparticles with four orthogonal single-particle analysis platforms. J. Extracell. Vesicles.

[bb0020] Asadujjaman M., Nam Y.R., Lee D.-E., Choi J.U., Kim S.H., Jang D.-J., Jee J.-P. (2025). Fusion nanoparticle system of extracellular vesicles and docetaxel-loaded liposomes: an innovative therapeutic strategy to enhance anticancer efficacy. J. Pharm. Investig..

[bb0025] Besse B., Charrier M., Lapierre V., Dansin E., Lantz O., Planchard D., Le Chevalier T., Livartoski A., Barlesi F., Laplanche A., Ploix S., Vimond N., Peguillet I., Thery C., Lacroix L., Zoernig I., Dhodapkar K., Dhodapkar M., Viaud S., Chaput N. (2016). Dendritic cell-derived exosomes as maintenance immunotherapy after first line chemotherapy in NSCLC. Oncoimmunology.

[bb0030] Bosch S., de Beaurepaire L., Allard M., Mosser M., Heichette C., Chretien D., Jegou D., Bach J.M. (2016). Trehalose prevents aggregation of exosomes and cryodamage. Sci. Rep..

[bb0035] Charoenviriyakul C., Takahashi Y., Nishikawa M., Takakura Y. (2018). Preservation of exosomes at room temperature using lyophilization. Int. J. Pharm..

[bb0040] Chaudhari A.P., Budayr O.M., Bonacquisti E.E., Kussatz C.C., Bannon M.S., Law K.J., Liu Y., Bolton M.L., Glassman P.M., Huang L., Nguyen J. (2026). The status of extracellular vesicles as drug carriers and therapeutics. Nat. Rev. Bioeng..

[bb0045] Chen M.Y., Cheng T.W., Pan Y.C., Mou C.Y., Chiang Y.W., Lin W.C., Hu C.J., Mou K.Y. (2025). Endotoxin-free outer membrane vesicles for safe and modular anticancer immunotherapy. ACS Synth. Biol..

[bb0055] Committee for Advanced Therapies (2026).

[bb0060] Daaboul G.G., Gagni P., Benussi L., Bettotti P., Ciani M., Cretich M., Freedman D.S., Ghidoni R., Ozkumur A.Y., Piotto C., Prosperi D., Santini B., Unlu M.S., Chiari M. (2016). Digital detection of exosomes by interferometric imaging. Sci. Rep..

[bib411] European Commission (2009). Commission Directive 2009/120/EC of 14 September 2009 amending Directive 2001/83/EC of the European Parliament and of the Council on the Community code relating to medicinal products for human use as regards advanced therapy medicinal products. Official Journal of the European Union L.

[bb0065] European Medicines Agency (2018).

[bb0070] European Medicines Agency (2025).

[bb0075] European Medicines Agency (2026). https://www.ema.europa.eu/en/human-regulatory-overview/marketing-authorisation/advanced-therapies-marketing-authorisation/advanced-therapy-classification.

[bb0080] European Medicines Agency, U.S. Food and Drug Administration (2021).

[bib412] European Parliament and Council of the European Union (2007). Regulation (EC) No 1394/2007 of the European Parliament and of the Council of 13 November 2007 on advanced therapy medicinal products and amending Directive 2001/83/EC and Regulation (EC) No 726/2004. Official Journal of the European Union L.

[bb0085] Frank J., Richter M., de Rossi C., Lehr C.M., Fuhrmann K., Fuhrmann G. (2018). Extracellular vesicles protect glucuronidase model enzymes during freeze-drying. Sci. Rep..

[bb0090] Geeurickx E., Tulkens J., Dhondt B., Van Deun J., Lippens L., Vergauwen G., Heyrman E., De Sutter D., Gevaert K., Impens F., Miinalainen I., Van Bockstal P.J., De Beer T., Wauben M.H.M., Nolte-’t-Hoen E.N.M., Bloch K., Swinnen J.V., van der Pol E., Nieuwland R., Hendrix A. (2019). The generation and use of recombinant extracellular vesicles as biological reference material. Nat. Commun..

[bb0095] Gelibter S., Marostica G., Mandelli A., Siciliani S., Podini P., Finardi A., Furlan R. (2022). The impact of storage on extracellular vesicles: a systematic study. J. Extracell. Vesicles.

[bb0100] Gimona M., Brizzi M.F., Choo A.B.H., Dominici M., Davidson S.M., Grillari J., Hermann D.M., Hill A.F., de Kleijn D., Lai R.C., Lai C.P., Lim R., Monguio-Tortajada M., Muraca M., Ochiya T., Ortiz L.A., Toh W.S., Yi Y.W., Witwer K.W., Lim S.K. (2021). Critical considerations for the development of potency tests for therapeutic applications of mesenchymal stromal cell-derived small extracellular vesicles. Cytotherapy.

[bb0105] Gorgens A., Corso G., Hagey D.W., Jawad Wiklander R., Gustafsson M.O., Felldin U., Lee Y., Bostancioglu R.B., Sork H., Liang X., Zheng W., Mohammad D.K., van de Wakker S.I., Vader P., Zickler A.M., Mamand D.R., Ma L., Holme M.N., Stevens M.M., El Andaloussi S. (2022). Identification of storage conditions stabilizing extracellular vesicles preparations. J. Extracell. Vesicles.

[bb0110] Hong C.M., Gangadaran P., Oh J.M., Rajendran R.L., Gopal A., Zhu L., Ahn B.C. (2021). Radioiodine labeling and in vivo trafficking of extracellular vesicles. Sci. Rep..

[bb0115] ICH Cell & Gene Therapy Discussion Group (2025).

[bb0120] ICH Q1 Expert Working Group (2026).

[bb0125] International Conference on Harmonisation of Technical Requirements for Registration of Pharmaceuticals for Human Use (1995).

[bb0130] International Conference on Harmonisation of Technical Requirements for Registration of Pharmaceuticals for Human Use (1999).

[bb0135] International Conference on Harmonisation of Technical Requirements for Registration of Pharmaceuticals for Human Use (2003).

[bb0140] International Conference on Harmonisation of Technical Requirements for Registration of Pharmaceuticals for Human Use (2004).

[bb0145] International Conference on Harmonisation of Technical Requirements for Registration of Pharmaceuticals for Human uUse (2008).

[bb0150] International Conference on Harmonisation of Technical Requirements for Registration of Pharmaceuticals for Human Use (2009).

[bb0155] International Conference on Harmonisation of Technical Requirements for Registration of Pharmaceuticals for Human Use (2012).

[bb0160] International Council for Harmonisation of Technical Requirements for Pharmaceuticals for Human Use (2023).

[bb0165] International Council for Harmonisation of Technical Requirements for Pharmaceuticals for Human Use (2023).

[bb0170] International Council for Harmonisation of Technical Requirements for Pharmaceuticals for Human uUse (2023).

[bb0175] International Council for Harmonisation of Technical Requirements for Pharmaceuticals for Human uUse (2023).

[bb0180] International Council for Harmonisation of Technical Requirements for Pharmaceuticals for Human Use (2023).

[bb0185] International Council for Harmonisation of Technical Requirements for Pharmaceuticals for Human Use (2025).

[bb0190] International Council for Harmonisation of Technical Requirements for Pharmaceuticals for Human Use (2025).

[bb0195] Kamerkar S., LeBleu V.S., Sugimoto H., Yang S., Ruivo C.F., Melo S.A., Lee J.J., Kalluri R. (2017). Exosomes facilitate therapeutic targeting of oncogenic KRAS in pancreatic cancer. Nature.

[bb0200] Khan A.A., Man F., Faruqu F.N., Kim J., Al-Salemee F., Carrascal-Minino A., Volpe A., Liam-Or R., Simpson P., Fruhwirth G.O., Al-Jamal K.T., de Rosales R.T.M. (2022). PET imaging of small extracellular vesicles via [(89)Zr]Zr(oxinate)(4) direct radiolabeling. Bioconjug. Chem..

[bb0205] Kuribayashi R. (2025).

[bb0210] Kusuma G.D., Li A., Zhu D., McDonald H., Inocencio I.M., Chambers D.C., Sinclair K., Fang H., Greening D.W., Frith J.E., Lim R. (2022). Effect of 2D and 3D culture microenvironments on mesenchymal stem cell-derived extracellular vesicles potencies. Front. Cell Dev. Biol..

[bb0215] Lai C.P., Mardini O., Ericsson M., Prabhakar S., Maguire C., Chen J.W., Tannous B.A., Breakefield X.O. (2014). Dynamic biodistribution of extracellular vesicles in vivo using a multimodal imaging reporter. ACS Nano.

[bb0220] Lazaro-Ibanez E., Faruqu F.N., Saleh A.F., Silva A.M., Tzu-Wen Wang J., Rak J., Al-Jamal K.T., Dekker N. (2021). Selection of fluorescent, bioluminescent, and radioactive tracers to accurately reflect extracellular vesicle biodistribution in vivo. ACS Nano.

[bb0225] Lee S.-K., Ha E.-S., Park H., Jeong J.-S., Shin C.Y., Kim J.-S., Jeong S.H., Kim M.-S. (2025). Lyophilization of pharmaceuticals: an overview and practical approaches for developing the lyophilization cycle. J. Pharm. Investig..

[bb0230] Lee J., Min J., Kim D., Choi Y., Park J., Kim M.-J., Park C., Jee J.-P., Park Y.-J., Shim G. (2026). Nanoparticle-based critical quality attributes in complex generic development: regulatory perspectives from PEGylated liposomal doxorubicin case study. J. Pharm. Investig..

[bb0235] Levy D., Jeyaram A., Born L.J., Chang K.H., Abadchi S.N., Hsu A.T.W., Solomon T., Aranda A., Stewart S., He X., Harmon J.W., Jay S.M. (2023). Impact of storage conditions and duration on function of native and cargo-loaded mesenchymal stromal cell extracellular vesicles. Cytotherapy.

[bb0240] Li Q., Li Y., Shao J., Sun J., Hu L., Yun X., Liuqing C., Gong L., Wu S. (2025). Exploring regulatory frameworks for exosome therapy: insights and perspectives. Health Care Sci..

[bb0245] Lyu N., Knight R., Robertson S.Y.T., Dos Santos A., Zhang C., Ma C., Xu J., Zheng J., Deng S.X. (2022). Stability and function of extracellular vesicles derived from immortalized human corneal stromal stem cells: a proof of concept study. AAPS J..

[bb0250] Mateescu B., Kowal E.J., van Balkom B.W., Bartel S., Bhattacharyya S.N., Buzas E.I., Buck A.H., de Candia P., Chow F.W., Das S., Driedonks T.A., Fernandez-Messina L., Haderk F., Hill A.F., Jones J.C., Van Keuren-Jensen K.R., Lai C.P., Lasser C., Liegro I.D., Nolte-’t Hoen E.N. (2017). Obstacles and opportunities in the functional analysis of extracellular vesicle RNA - an ISEV position paper. J. Extracell. Vesicles.

[bb0255] Moon H., Kim J., Bae G., Choi W., Jung S.H., Lee S., Lim K., Na J.H., Choi J.U., Kim J.-H., Lee S. (2025). Cell membrane-camouflaged nanoparticles: selection strategy in incurable disease treatment. J. Pharm. Investig..

[bb0260] National Institute of Food and Drug Safety Evaluation (2024).

[bb0265] Park H., Lee J., Kang J.-W., Min J.-Y., Lee J., Hong J., Shim G. (2025). Cationic lipid nanoparticles for nucleic acid delivery: microfluidics versus thin film hydration. J. Pharm. Investig..

[bb0270] Poudel S., Nelson D.L., Yen J.H., Wang Y., Zhang H., He Z., Green A.B., Veerland W.N., Cleveland T.E.t., Lehman S.E., Benkstein K.D., Nelson B.C., Wang L. (2025). Intermethod characterization of commercially available extracellular vesicles as reference materials. Biomolecules.

[bb0275] Puzar Dominkus P., Stenovec M., Sitar S., Lasic E., Zorec R., Plemenitas A., Zagar E., Kreft M., Lenassi M. (2018). PKH26 labeling of extracellular vesicles: characterization and cellular internalization of contaminating PKH26 nanoparticles. Biochim. Biophys. Acta Biomembr..

[bb0285] Rikkert L.G., Nieuwland R., Terstappen L., Coumans F.A.W. (2019). Quality of extracellular vesicle images by transmission electron microscopy is operator and protocol dependent. J. Extracell. Vesicles.

[bb0290] Royo F., Cossio U., Ruiz de Angulo A., Llop J., Falcon-Perez J.M. (2019). Modification of the glycosylation of extracellular vesicles alters their biodistribution in mice. Nanoscale.

[bb0295] Simon C.G., Borgos S.E., Calzolai L., Nelson B.C., Parot J., Petersen E.J., Roesslein M., Xu X., Caputo F. (2023). Orthogonal and complementary measurements of properties of drug products containing nanomaterials. J. Control. Release.

[bb0300] Smyth T., Kullberg M., Malik N., Smith-Jones P., Graner M.W., Anchordoquy T.J. (2015). Biodistribution and delivery efficiency of unmodified tumor-derived exosomes. J. Control. Release.

[bb0305] Subcommittee on Therapeutic Products Based on Extracellular Vesicles (EVs) Including Exosomes (2023).

[bb0310] Susa F., Limongi T., Borgione F., Peiretti S., Vallino M., Cauda V., Pisano R. (2023). Comparative studies of different preservation methods and relative freeze-drying formulations for extracellular vesicle pharmaceutical applications. ACS Biomater. Sci. Eng..

[bb0315] Takakura Y., Hanayama R., Akiyoshi K., Futaki S., Hida K., Ichiki T., Ishii-Watabe A., Kuroda M., Maki K., Miura Y., Okada Y., Seo N., Takeuchi T., Yamaguchi T., Yoshioka Y. (2024). Quality and safety considerations for therapeutic products based on extracellular vesicles. Pharm. Res..

[bb0320] Tertel T., Dittrich R., Arsene P., Jensen A., Giebel B. (2023). EV products obtained from iPSC-derived MSCs show batch-to-batch variations in their ability to modulate allogeneic immune responses in vitro. Front. Cell Dev. Biol..

[bb0325] Trenkenschuh E., Richter M., Heinrich E., Koch M., Fuhrmann G., Friess W. (2022). Enhancing the stabilization potential of lyophilization for extracellular vesicles. Adv. Healthc. Mater..

[bb0330] U.S. Food and Drug Administration (2011).

[bb0335] U.S. Food and Drug Administration (2019).

[bb0340] U.S. Food and Drug Administration (2023). https://www.fda.gov/inspections-compliance-enforcement-and-criminal-investigations/warning-letters/kimera-labs-inc-649343-09012023.

[bb0345] Van Delen M., Derdelinckx J., Wouters K., Nelissen I., Cools N. (2024). A systematic review and meta-analysis of clinical trials assessing safety and efficacy of human extracellular vesicle-based therapy. J. Extracell. Vesicles.

[bb0350] van der Pol E., Coumans F.A., Grootemaat A.E., Gardiner C., Sargent I.L., Harrison P., Sturk A., van Leeuwen T.G., Nieuwland R. (2014). Particle size distribution of exosomes and microvesicles determined by transmission electron microscopy, flow cytometry, nanoparticle tracking analysis, and resistive pulse sensing. J. Thromb. Haemost..

[bb0355] Verma N., Arora S. (2025). Navigating the global regulatory landscape for exosome-based therapeutics: challenges, strategies, and future directions. Pharmaceutics.

[bb0360] Vestad B., Llorente A., Neurauter A., Phuyal S., Kierulf B., Kierulf P., Skotland T., Sandvig K., Haug K.B.F., Ovstebo R. (2017). Size and concentration analyses of extracellular vesicles by nanoparticle tracking analysis: a variation study. J. Extracell. Vesicles.

[bb0365] Webber J., Clayton A. (2013). How pure are your vesicles?. J. Extracell. Vesicles.

[bb0370] Wei W., Wu X., Wang L., Yu Y., Yang L., Zhang C., Wang D. (2026). The global clinical landscape of human-derived extracellular vesicles: an analysis based on ClinicalTrials.gov (2010-2025). Clin. Exp. Med..

[bb0375] Welsh J.A., Van Der Pol E., Arkesteijn G.J.A., Bremer M., Brisson A., Coumans F., Dignat-George F., Duggan E., Ghiran I., Giebel B., Gorgens A., Hendrix A., Lacroix R., Lannigan J., Libregts S., Lozano-Andres E., Morales-Kastresana A., Robert S., De Rond L., Jones J.C. (2020). MIFlowCyt-EV: a framework for standardized reporting of extracellular vesicle flow cytometry experiments. J. Extracell. Vesicles.

[bb0380] Welsh J.A., van der Pol E., Bettin B.A., Carter D.R.F., Hendrix A., Lenassi M., Langlois M.A., Llorente A., van de Nes A.S., Nieuwland R., Tang V., Wang L., Witwer K.W., Jones J.C. (2020). Towards defining reference materials for measuring extracellular vesicle refractive index, epitope abundance, size and concentration. J. Extracell. Vesicles.

[bb0385] Welsh J.A., Arkesteijn G.J.A., Bremer M., Cimorelli M., Dignat-George F., Giebel B., Gorgens A., Hendrix A., Kuiper M., Lacroix R., Lannigan J., van Leeuwen T.G., Lozano-Andres E., Rao S., Robert S., de Rond L., Tang V.A., Tertel T., Yan X., van der Pol E. (2023). A compendium of single extracellular vesicle flow cytometry. J. Extracell. Vesicles.

[bb0390] Welsh J.A., Goberdhan D.C.I., O’Driscoll L., Buzas E.I., Blenkiron C., Bussolati B., Cai H., Di Vizio D., Driedonks T.A.P., Erdbrugger U., Falcon-Perez J.M., Fu Q.L., Hill A.F., Lenassi M., Lim S.K., Mahoney M.G., Mohanty S., Moller A., Nieuwland R., Witwer K.W. (2024). Minimal information for studies of extracellular vesicles (MISEV2023): from basic to advanced approaches. J. Extracell. Vesicles.

[bb0395] Wiklander O.P., Nordin J.Z., O’Loughlin A., Gustafsson Y., Corso G., Mager I., Vader P., Lee Y., Sork H., Seow Y., Heldring N., Alvarez-Erviti L., Smith C.I., Le Blanc K., Macchiarini P., Jungebluth P., Wood M.J., Andaloussi S.E. (2015). Extracellular vesicle in vivo biodistribution is determined by cell source, route of administration and targeting. J. Extracell. Vesicles.

[bb0400] Xu G., Jin J., Fu Z., Wang G., Lei X., Xu J., Wang J. (2025). Extracellular vesicle-based drug overview: research landscape, quality control and nonclinical evaluation strategies. Signal Transduct. Target. Ther..

[bb0405] Yuana Y., Koning R.I., Kuil M.E., Rensen P.C., Koster A.J., Bertina R.M., Osanto S. (2013). Cryo-electron microscopy of extracellular vesicles in fresh plasma. J. Extracell. Vesicles.

[bb0410] Zhang H., Freitas D., Kim H.S., Fabijanic K., Li Z., Chen H., Mark M.T., Molina H., Martin A.B., Bojmar L., Fang J., Rampersaud S., Hoshino A., Matei I., Kenific C.M., Nakajima M., Mutvei A.P., Sansone P., Buehring W., Lyden D. (2018). Identification of distinct nanoparticles and subsets of extracellular vesicles by asymmetric flow field-flow fractionation. Nat. Cell Biol..

